# Ultrafast Imaging of Physiological Brain Pulsations With Magnetic Resonance Encephalography—From Noise to Predictive Clinical Biomarker

**DOI:** 10.1002/nbm.70092

**Published:** 2025-07-08

**Authors:** Vesa Kiviniemi, Heta Helakari, Lauri Raitamaa, Niko Huotari, Zalan Rajna, Matti Järvelä, Ahmed Elabasy, Johanna Tuunanen, Valter Poltojainen, Tommi Väyrynen, Timo Tuovinen, Janne Kananen, Vesa Korhonen

**Affiliations:** ^1^ Oulu Functional NeuroImaging HST/Oulu University Oulu Finland; ^2^ Diagnostics/MRC Oulu University Hospital Oulu Finland; ^3^ Biocenter Oulu University of Oulu Oulu Finland

## Abstract

Over the past decade, novel in vivo imaging techniques have revealed that physiological pulsations drive the transport of brain solutes and that impairment of fluid flow precedes certain neuropathologies. Although the pioneering investigations on brain solute transport mechanisms mainly employed imaging of exogenous tracers, novel advanced ultrafast functional MRI sequences enable critical sampling of propagating physiological pulsations driving the brain fluids devoid of aliased mixing of signals. In this review, we summarize the emerging magnetic resonance encephalography (MREG) technique, beginning with a historical perspective and physiological background of the phenomena of brain pulsatility as measured in the parenchyma and cerebrospinal fluid (CSF). We give a detailed account of how functional contrast mechanisms evident in the T2(*)‐weighted MREG signal enable the simultaneous mapping of three distinct physiological signals. Our narrative review continues with an account of signal analysis and methodological considerations arising from 12 years of experience in ultrafast brain scanning. Our review concludes with a presentation of how sleep‐related physiological changes in the driving pulsations influence solute transport in a healthy brain and our perspective on the potential of these pulsations as emerging biomarkers for predictive, diagnostic, and treatment monitoring in the context of Alzheimer's disease and other central nervous system (CNS) conditions.

## Graphical abstract

Human brain pulsations shown in this picture drive both blood and CSF flow. These pulsations can now be accurately detected with ultrafast MREG imaging. The ultrafast imaging offers early detection of abnormal brain pulsations that can precede tissue degeneration in Alzheimer's disease or predict mortality in brain lymphoma treatment.

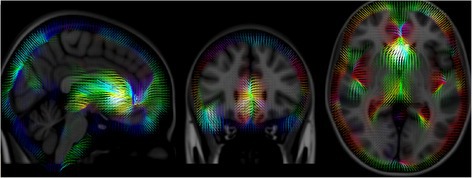

**Abbreviations**
[de]oxyHb: [de]oxygenated hemoglobin
AAN: ascending arousal network
ad
: Alzheimer's disease
AQP4: aquaporin 4
AUC: area under curve
BBB: blood–brain barrier
BOLD: blood oxygenation level dependent
BOLD_CV_
: coefficient of variation in BOLD signal
CHE: cardiohemodynamic envelope
CSF: cerebrospinal fluid
EEG: electroencephalography
EPI: echo planar imaging
EVI: echo volumetric imaging
IPAD: intramural periarterial drainage
ISF: interstitial space fluid
MPM: multiphoton microscopy
MREG: magnetic resonance encephalography
MRI: magnetic resonance imaging
MTS: mesial temporal lobe sclerosis
NT1: narcolepsy Type I
OVOC: one voxel one coil
PCNSL: primary central nervous system lymphoma
PCNSL: primary central nervous system lymphoma
SSFP: steady state free precession
VLF: very low frequency

## 1 History of Mapping Brain Pulsations

Ancient physicians were aware of cerebral pulsations from their observations in patients with open skull injuries, but a consideration of their functional significance may date to the Turin physiologist Angelo Mosso's 1880 description of increased brain pulsatility during performance of cued visual and cognitive tasks in patients with skull defects following neurosurgical procedures [1]. Only a decade later, the Cambridge physiologists Roy and Sherrington reported pulsatile responses during electric brain stimulus in an animal experiment, where “… in our experience especially frequently in such experiments … the brain expands with each rise of the blood‐pressure and contracts with each successive fall” [2]. At the turn of the 20th century, the Jena psychiatrist Hans Berger described three forms of intracranial pressure pulsations: “eine pulsatorische, eine respiratorische und vasomotorische Bewegung” [3] while pursuing his studies of pulsatile brain activity that ultimately led to his invention of electroencephalography (EEG) in 1924. Importantly, these pioneering studies were all performed using analog data sampling methods that enabled the inherent separation of simultaneously occurring physiological brain pulsations without aliasing, a phenomenon often arising in wave sampling, when high‐frequency signals appear as lower frequency signals because of insufficient sampling rate.

**FIGURE 1:**
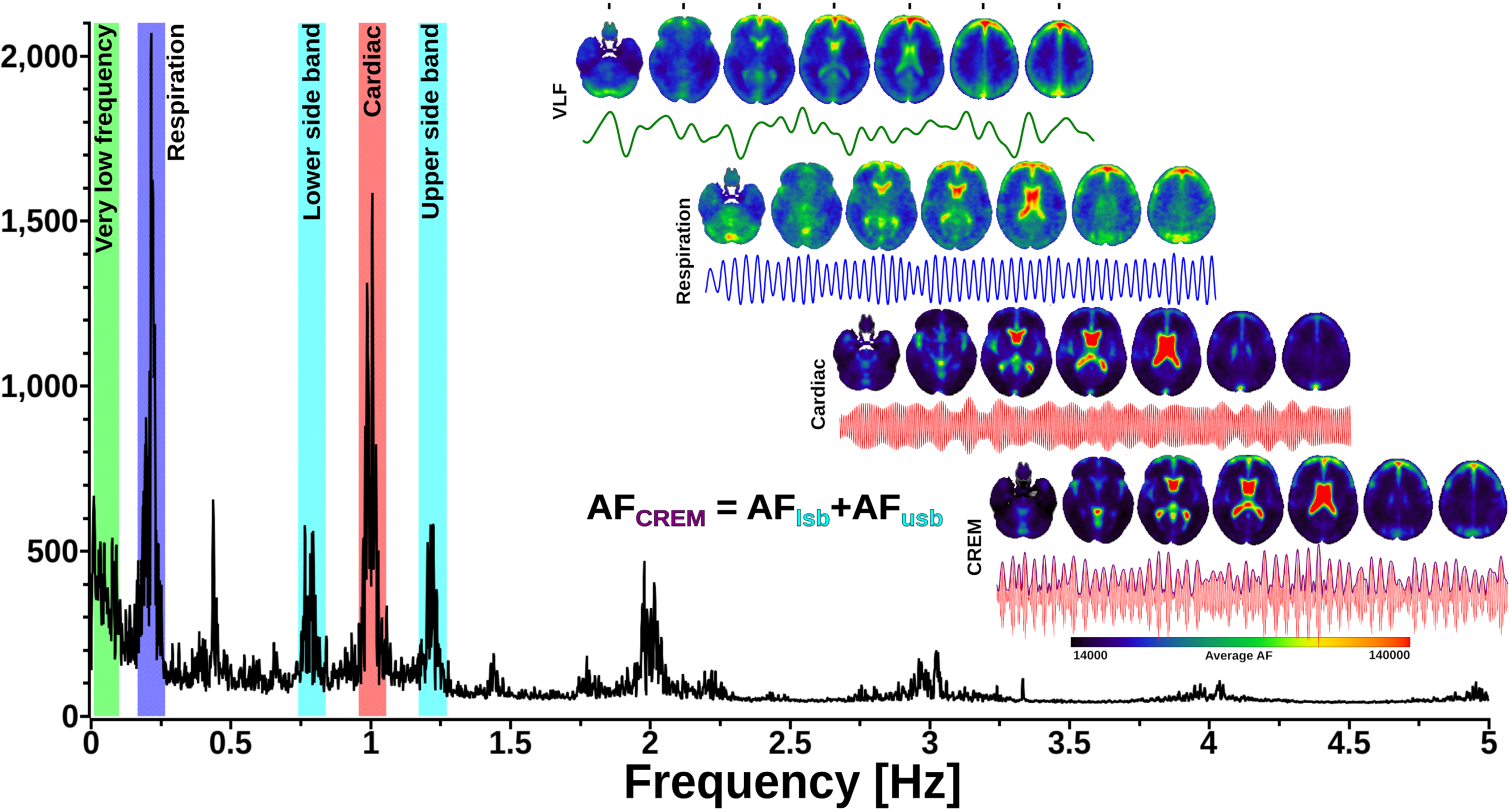
A representative whole‐brain (a.k.a. global signal) amplitude spectrum extending to 5 Hz of fluctuations in the MREG BOLD signal, along with inserts showing the respective amplitude maps for the three physiological brain pulsation signals. The very low‐frequency (VLF < 0.1 Hz) vasomotor wave band is shown in the green sector, the circa 0.23 Hz respiratory pulsation band in lilac blue, and the 1 Hz cardiovascular pulsation band in red, along with the 1st, 2nd, 3rd, and 4th harmonic cardiac peaks. Importantly, MREG also enables the detection of modulatory heterodyne sideband peaks, here lying at 0.23 Hz on either side of the red principal cardiac peak. These signals reflect the amplitude modulation by respiratory pulsations on cardiovascular pulsations (CREM) in the brain, which can also be visualized as the blue envelope laid over the cardiovascular pulsation signal in the insert to the right, as presented in [4]. We note the admirably high physiological signal to thermal noise ratio (SNR_P/T_) of the respiratory (200) and cardiovascular (160) pulsations relative to the baseline thermal noise level, exemplifying their clear differentiation in the absence of aliasing over the VLF signal seen in slower fMRI. For 3D video depictions of each brain pulsation signal change in standard MNI space, also see 3D Videos [Link], [Link], [Link], [Link].

The presence of physiological pulsation bands is abundantly clear in the MREG spectral analysis as shown in Figure 1, but it may not be immediately apparent how these propagate to intracranial hydrodynamics of brain water in the various compartments of the intracranial space and spinal canal. According to the Monro–Kellie doctrine, any change in the volume of one of the brain fluid compartments, i.e., blood, cerebrospinal fluid (CSF), interstitial fluid (ISF), or intracellular volume, must be met with a compensatory change in the other compartments, given the fixed intracranial volume and the incompressibility of water. Therefore, the continuous physiological pulsations in the arterial and venous blood volumes drive the convention of the CSF and ISF.

In conventional understanding, the CSF serves to cushion the brain from head impacts, but there is growing appreciation of its role in mediating whole CNS homeostasis. The recently described paravascular solute convection mechanism, commonly known as the g (lial)lymphatic pathway, may utilize the ubiquitous hydrodynamic CSF pulsations as drivers from the convection of neuroactive substances together with soluble metabolic waste products toward lower resistance. In the original glymphatic model, solute convection follows along CSF conduits from the subarachnoid space into peri*arterial* Virchow–Robins spaces, then mixing with the ISF inside brain tissue, and ultimately exiting the brain along peri*venous* spaces [5, 6]. Although the polarized astroglial expression of aquaporin 4 (AQP4) water channels likely contributes vitally to the conduction of I/CSF hydrodynamic pulsations as a modulator of interastrocytic cleft size, there remain a few poorly understood aspects of this pathway for bulk flow of ISF and metabolite clearance in the brain.

In this narrative review of physiological pulsations in the CNS, we place our main focus on brain tissue, since current MREG methods are inadequate for investigating these phenomena deep in the spinal cord and spinal canal CSF. Both at the macroscopic and microscopic levels, cardiovascular pulsations in the brain predominate close to major cerebral arteries, subarachnoid (peri)arterial structures, and to a somewhat lesser extent in the sagittal sinus and central CSF ventricles [4, 7, 8, 9]. Macroscopically, cardiovascular pulsatility is the main driver of CSF flow in the cerebral aqueduct and through the foramen magnum, which occupies the space between the spinal canal and the intracranial volume [4, 9, 10, 11, 12, 13]. Among the three physiological pulsations, respiration band dominates in (peri)venous structures [8] but becomes more evident in CSF of the spinal cord and foramen magnum during deep breath taking [10, 11, 12, 13, 14]. The respiratory and vasomotor pulsations both predominate over cardiovascular pulsations within the cortical grey matter, whereas respiratory pulsations predominate in the white matter, deep nuclei, cerebellum, and brain stem in the living human brain [4, 9, 15, 16].

## 2 Novel Physiological Mechanisms

In a pioneering 1985 PET study, Fox and Raichle showed an excessive hyperemic oxygenation increase exceeding local glucose consumption in the human visual cortex upon exposure to a visual stimulus [17], which defies physiological explanation to this day. Drawing upon notes by Michael Faraday and early work of Linus Pauling on the para/diamagnetic properties of blood [18], in 1990, Seiji Ogawa established the use of repetitive functional magnetic resonance imaging (fMRI) in a protocol sensitive to the blood flow and oxygenation level of hemoglobin in the brain [19]. Serial T2*‐weighted fMRI scans conducted originally every few seconds confirmed the discrepant increase in regional blood oxygenation in activated cortical regions, which Ogawa designated as the blood oxygenation level dependent (BOLD) signal [19]. In a matter of 2 years, several research groups discovered that a regional activation in the human brain leads to an increase in BOLD contrast with a latency of 3–5 s, depending on the activated area [20, 21, 22].

**FIGURE 2:**
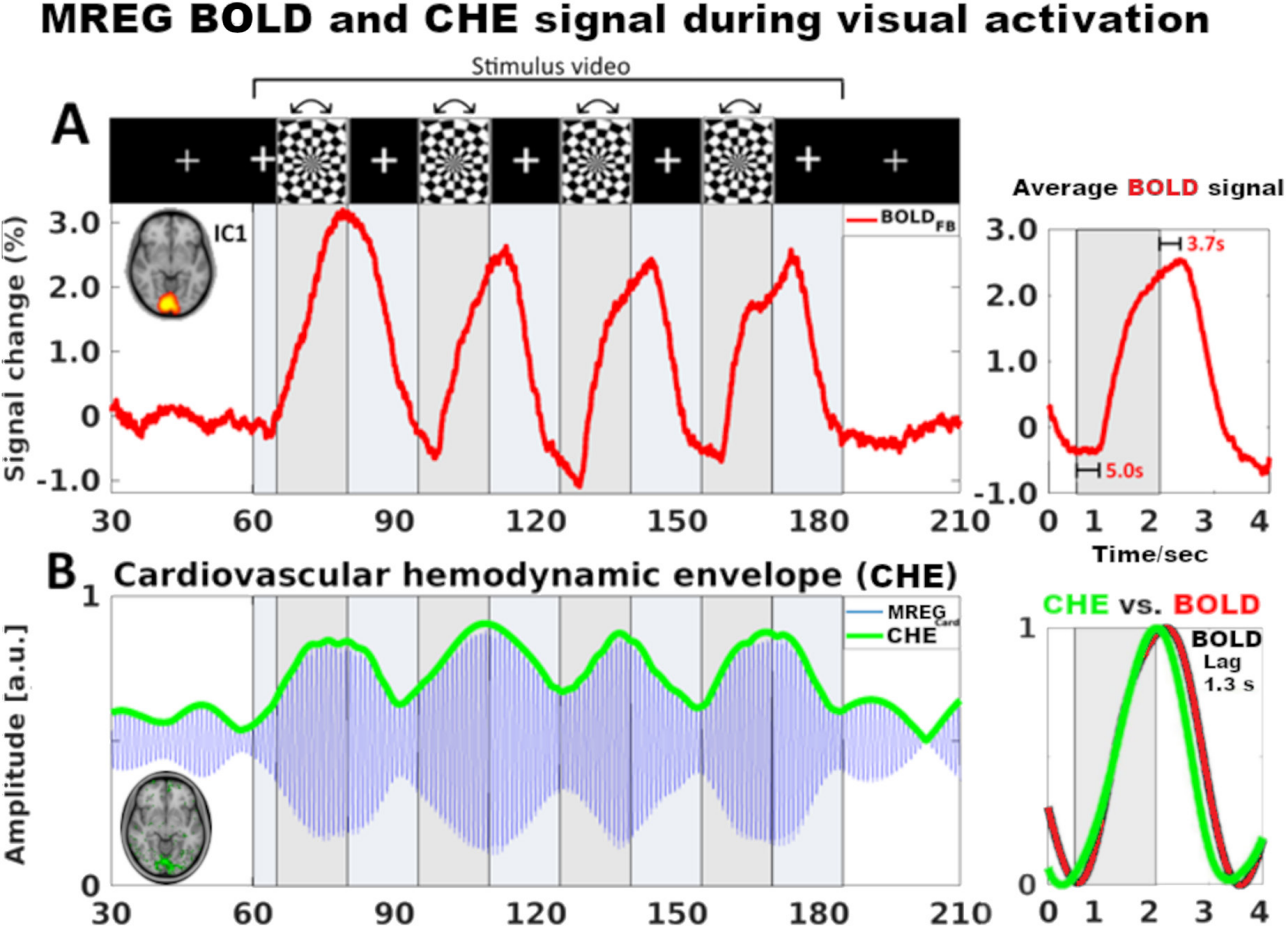
Ultrafast T2* signal scanning with MREG sequence enables detection of an increase in the arterial pulsatility prior to the classical venous BOLD response. (A) The classic susceptibility‐based BOLD (red signal) changes follow visual stimulation of rotating checkerboard activation. (B) Arterial cardiohemodynamic envelope (CHE, in green) laid over the blue cardiovascular signal of the same MREG data band‐passed to the individual's 1‐Hz cardiovascular signal. The CHE reflects neurovascular coupled dilatations of arteries based on increased cardiovascular impulse amplitude, much as in the early work of Mosso [1]. The green CHE envelope precedes the classical red BOLD signal by a mean of 1.3 s, as the arteries first dilate to increase the pulsatile flow into draining venous vessels later. The arterial dilation may also originate from closer to the activation site, as the classical susceptibility‐based BOLD contrast originates from venules downstream from the activated area (modified from [25]).

Current literature holds that activated neurons stimulate the local neurovascular unit, which triggers a dilation of regional precapillary arteriole sphincters. Although many believe that neuro‐astrocytic interaction triggers this activation hyperemia, actually, Nelson and colleagues have shown that the capillaries themselves sense the neuronal activation‐related [K^+^] increase that sends an inward activation potential over endothelial cell gap junctions to induce a upstream vasodilation. This relaxation of periarterial smooth muscle leads to vasodilation and consequent increases in pulsatile blood flow, with a response delay of 1–2 s [23, 24, 25, 26]. The enhanced inflow of oxygenated blood balloons the cortical venules and veins draining the activated area some 2–3 s after vasodilation, depending on the activated area [25, 27, 28], cf. Figure 2A–B. In terms of the MR contrast mechanism, the transiently increased volume of oxygenated blood in veins reduces the local concentration of paramagnetic deoxyhemoglobin [28]. The concomitant spike in venous blood oxygenation volume reduces dephasing of regional (peri)vascular water proton spins in fMRI data, which manifests as an increase in the susceptibility‐weighted T2* BOLD signal intensity level downstream from the activated areas [20, 21, 22, 28].

An important shortcoming of BOLD fMRI has previously been the low temporal resolution. In practice, acquiring images at intervals of a few seconds cannot adequately capture all relevant forms of brain physiological pulsations and, importantly, result in unrecoverable aliasing of the faster cardiorespiratory signal over the slower vasomotion‐induced BOLD signal changes [15]. This aliasing phenomenon is especially troubling in the cerebral cortex, where respiratory power and vasomotor BOLD signal power are roughly equal [4].

Faster BOLD data sampling that fulfills the minimum Nyquist theorem requirement of > 2 Hz sampling rate can remedy the problem of nonaliased detection of the cardiovascular pulsations [15]. Indeed, recent advances in ultrafast 3D fMRI sequences such as MREG, VEPI, GIN, and INI offer 20–40 times faster T2*‐weighted BOLD data sampling compared with older echo planar imaging (EPI) scans. This enables robust separation of all three physiological brain pulsation mechanisms, i.e., vasomotor, respiratory, and cardiovascular [9, 29, 30, 31]. Importantly, these high sampling rates improve the resolution in both frequency and time domains, which enables the detection of several new physiological phenomena of brain hydrodynamics.

For example, the modulations between physiological brain pulsations are now detectable in 3D, in addition to the three classical brain pulsations. These novel modulations may stem from direct physiological interactions between pulsations such as the cardiorespiratory pulsations (CREM; Figure 1), where the respiratory brain pulsations modulate the amplitude of the cardiovascular brain pulses in brain tissue, especially in the CSF spaces [4]. The CREM modulation travels as a wave throughout the intracranial space but has a unique spatiotemporal pattern distinct from that of respiratory pulses (cf. 3D videos of CREM and respiration in the Supporting Information).

**FIGURE 3:**
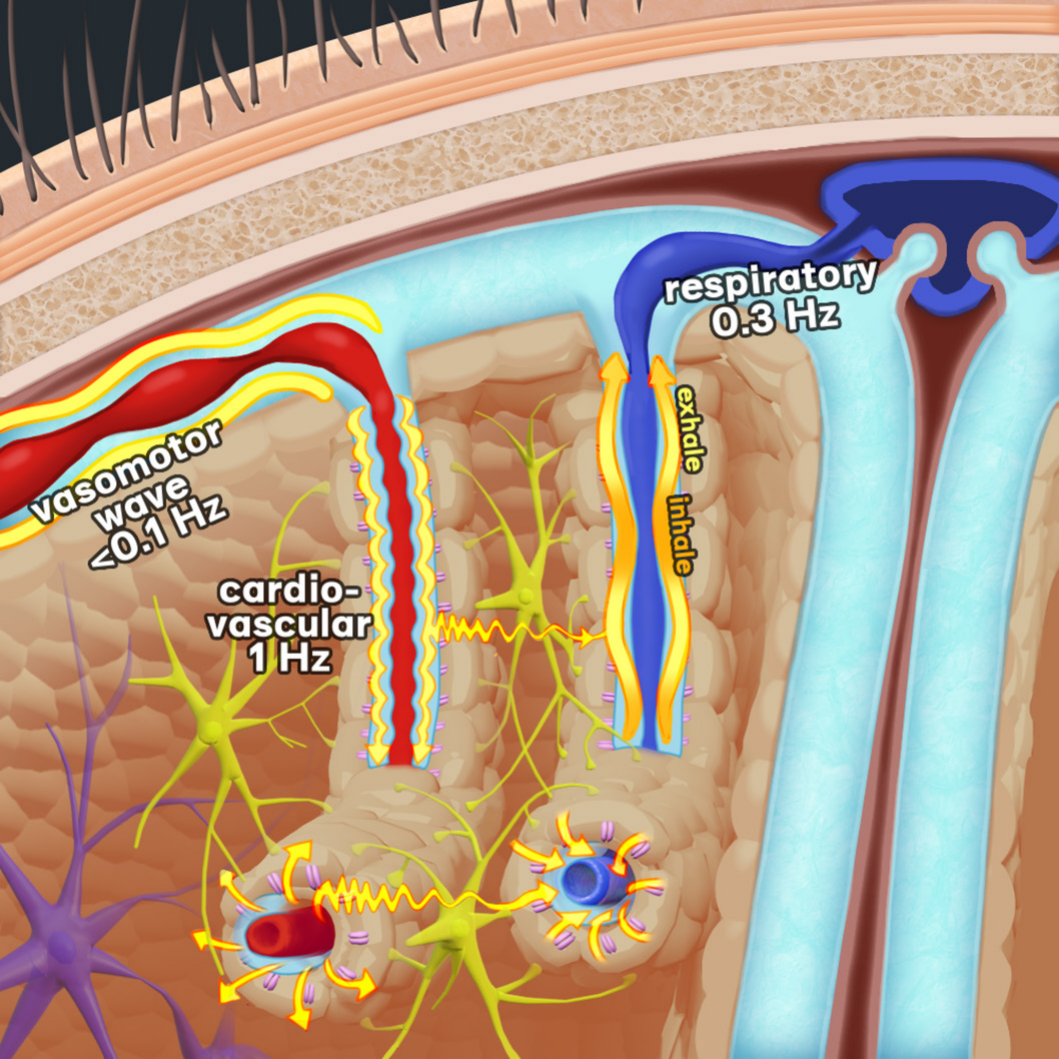
Schematic illustration of how physiological brain pulsations may propagate in brain tissue. Resistance changes from vasomotor waves in the smooth muscles encircling arterioles and larger arteries control cerebral blood flow, while also modulating the cardiorespiratory pulsation amplitudes downstream from the arterioles, and moving solutes by a pumping action [32, 33, 34]. Arterial pulsation pushes blood along the vessel lumen and CSF along the periarterial Virchow–Robin space, according to the recently described glymphatic solute transport mechanism. In this mechanism, water enters the astrocytes via aquaporin 4 (AQP4) channels and CSF with solutes into the interstitium via astrocyte endfeet clefts. The arterial impulses induce water spin phase coherence changes that transiently drop MR signal intensity during the systolic phase of the cardiac cycle, whereupon the spin phase coherence returns and thereby increases the signal inside blood vessels and in perivascular spaces. Deep inhalation is especially apt to draw venous blood from the cortical veins and promote compensatory inward CSF flow to the perivenous space, countering the decline in venous blood volume. During exhalation, the intrathoracic pressure attenuates flow in cerebral veins. The ongoing perfusion from the capillaries then balloons the cortical veins with deoxygenated blood. The ballooning venous walls then extrude the CSF along with its solutes from the perivenous space. This results in a pumping effect within the perivenous space over respiratory cycles, as described elsewhere in detail [11, 13, 35]. See also 3D videos in the Supporting Information.

Another novel detectable form of modulation is driven by vasomotor dilatations in response to neuronal activation, as described above [25], cf. Figures 1 and 3. Local vasodilations of arterioles lead to episodic regional hyperremia, during which the relaxed vessels start to pulsate more than in their contracted state prior to vasodilation, as first shown by Mosso et al. in 1880 by analogue brain pulsation detection via cranial defect, and later confirmed by Kucewizc et al. in a fast‐sampling ultrasound study [36].

As a local vasodilation event precedes the blood oxygenation changes in veins downstream from the activated brain tissue, placing an envelope over successive cardiohemodynamic impulses (CHE) enables the detection of regional activity as increased arterial pulsation in some 1.3 s prior to the classical BOLD signal closer to the activated area itself [25]. Fast MREG recordings thus noninvasively provide evidence supporting the original finding of Musso, where brain activation led to a vasodilatory increase of arterial pulsatility (a.k.a. CHE) and likewise a blood volume and oxygenation (a.k.a. BOLD) signal increase in the aftermath of neuronal activation, cf. Figure 2.

## 3 Cardiovascular MREG Signal

The MREG signal, like any T2*‐weighted signal, mainly measures resonance from the abundant water protons in the brain and CSF. Since the 1980s, researchers have known that fast cardiovascular impulses in and around arteries introduce spin phase changes and steady‐state spin‐coherence perturbations in gradient‐recalled sequences [37, 38].

Ultrafast 3D VEPI and MREG sequences have both detected similar drops in arterial T2* signal intensity upon the arrival of cardiovascular impulses that traverse initially along the arterial vasculature and then extend to the periarterial space. The large fluid volumes of the CSF and sagittal sinus also have strong cardiac pulsation power because of the compensatory flow mechanisms articulated in the Monro–Kellie doctrine [9, 31]. The arterial signal drop is strongest near cerebral arteries and in central CSF spaces, but is less powerful than the vasomotor VLF and respiratory brain pulsations within the human brain grey and white matter [4].

Microscopic studies in rodent brain have shown that parenchymal cardiovascular pulsatility is also the main contributor to periarterial CSF water convection in the cortical vessels [39], in a functional‐anatomic pathway designated as the glymphatic system. As discovered by Nedergaard in 2012, these cardiovascular pulsations play a key role in maintaining brain tissue homeostasis by driving the CSF‐mediated convection of solutes through the brain parenchyma as mediated by AQP4 water channels and small pores between the astrocytic endfeet that sheath the BBB glia limitans [39, 40]. The amplitude of cardiovascular brain pulsations is directly proportional to the convective efficiency for transporting CSF solutes along perivascular CSF conduits [7].

As presented above, neuronal activation leads to relaxation of vasomotor tone in the neurovascular unit and also increases the amplitude of the low‐frequency envelope of cardiovascular pulsatility in activated areas some 1.3 (±2.2) seconds before BOLD response, cf. in Figure 2B). The pulsatile nature of blood flow has itself been shown to increase nitric oxide (NO)–mediated vasodilation, which further facilitates the downstream vasodilatory brain responses in the vascular trees [41]. Increased arterial pulsation further augments the metabolite flow in perivascular areas during neuronal activation [7, 42].

## 4 Respiratory MREG Signal

In all T2*‐weighted fMRI data, respiration induces waves in the detected BOLD signal that propagate along venous tracks: Acquisition of BOLD data at different sampling rates reveals that deeper voluntary inhalations increase the BOLD signal in the cerebral cortex and notably in the underlying white matter, whereas exhalation decreases the signal [4, 9, 43, 44, 45]. The respiratory brain tissue MREG signal is dominated by the classical venous susceptibility BOLD effect described above. Inhalation (especially deep inhalations) reduces the volume of deoxygenated blood in veins via transmission of intrathoracic negative pressure through the venous sinuses. Increased venous outflow then increases the T2*‐weighted MREG signal in the parenchyma. In accordance with the Monro–Kellie doctrine, the reduced venous blood volume is further replaced by inflowing CSF in the perivascular spaces [10, 11, 14, 46, 47, 48, 49]. The consequently increasing water volume in the perivascular CSF spaces increases the MREG signal with T2 effects.

Exhalation has somewhat opposite effects; there is a slowing of venous flow because of counter‐pressure inside the thorax. The veins then balloon with deoxygenated blood, which increases water proton dephasing and results in reduced T2* signal intensity. At the same time, the ballooning veins empty the perivascular CSF volume, which reduces the T2 effects even further. This cycle repeats in phase with the respiratory cycle, cf. Figure 3 and the 3D respiratory video in the Supporting Information.

Venous outflow from the brain has another interesting characteristic in relation to its propagation through the only incompressible veins in the body—the dural venous sinuses. All other veins in the body collapse under negative pressures, whereas within the dura, the structures surrounding venous sinuses remain open to mediate the negative pressures during inhalation into the subarachnoid space [10, 50]. Furthermore, this negative pressure feature within dural sinuses seems to be connected to the accumulation of solutes close to the brain vertex next to the dural lymphatic vessels, which occurs only next to venous sinuses [12]. This mechanism is also bound to affect the perivenous CSF space in the cortex, which, together with counter‐phase venous blood volume changes, creates a perivenous CSF pump, cf. Figure 3. Interestingly, the MREG technique is fit to detect sleep‐dependent changes in the coherence of arterial vasomotion (CHE) and the synchronization of venous vasomotor BOLD waves of < 0.1 Hz in these parasagittal areas, thus presenting a mechanism whereby solute efflux may increase during the sleep state [51].

The he glymphatic solute efflux transport, a.k.a. the slow convection of tracers injected below the cerebellum into paraspinal CSF spaces into the mammalian brain, seems to distribute the CSF back into the cortical CSF areas and grey matter, leaving the deeper structures like white matter, deep nuclei, and brain stem devoid of solute transportation [12, 52]. In our experience, the dominant pulsation driving the I/CSF in these deeper structures is the ubiquitous respiratory pulsation mediated via (peri)venous fluid dynamics.

## 5 Vasomotor Waves in MREG Signal

The first report of spontaneous vasomotor waves dates to Stephen Hales' 1733 study of continuous blood pressure measurement during exsanguination of a horse. In contemporary view, there are two characteristic types of these pressure waves: the slower, local arterial myocytic 0.03 Hz Mayer waves and the somewhat faster 0.1 Hz Traube–Hering waves stemming from slow gain in the heart–lung blood flow control. Not only present in systemic blood pressure recordings, such waves are also traceable in isolated blood vessels, being driven by the intrinsic ability of smooth muscle cells to undergo periodic depolarization [53, 54, 55]. The frequency of these vasomotor waves increases from large arteries toward the smaller arterioles and also blood pressure increases the frequency and reduces the amplitude [56]. Pioneering fMRI reports of spontaneous functional connectivity in the late 1990s drew a relation between the functionally connected VLF fluctuations with slow vasomotor waves [57, 58].

Complementing these intrinsic vascular waves, brain vasculature and hydrodynamics respond to whole‐body rhythms on the scale of hours, including the circadian rhythm, hormonal control mechanisms (e.g., renin‐angiotensin), and gastrointestinal blood volume changes. The multiple factors regulating blood flow and pressure waves act simultaneously, with summation in the measured signal in the manner of 1/f‐type fractal behavior, where fast changes in one factor may be of relatively small amplitude, but longer‐lasting changes involving multiple control mechanisms are of greater amplitude [54, 55]. A clear‐cut identification of unique isolated waves may be difficult because of their interaction with other control mechanisms. Thus, researchers tend to present their results in different frequency bands, depending on the underlying physiological state or pathology in a way highly like EEG.

In general, flow resistance controls the magnitude of regional cerebral blood flow (CBF), where the caliber of a given blood vessel varies according to the vasomotor tone of the myocytes encircling the arterial walls. Thus, CBF reflects net fluctuations in vasomotor tone under the integrated control of several systemic and regional control factors. This phenomenon of vasomotor regulation underlies the activation‐induced regional hyperemia driving BOLD signal changes in response to neuronal activation [19, 20, 28], cf. Figure 2.

In the awake condition, neuronal activity is tightly coupled to regional CBF during the performance of cued task activations [20] and in response to spontaneous brain activity fluctuations [57] within functionally connected, independent brain networks [57, 59, 60, 61, 62]. In addition to functionally connected standing activity waves comprising some 42 independent functional units [63, 64], the brain also hosts slow propagating vasomotor waves that traverse across the borders of independent functional networks and into CSF spaces [9, 63, 65, 66, 67]; even functionally connected white matter tracks can exhibit spatially oriented low‐frequency signals [68].

## 6 Sleep Physiology

As presented above, CBF in the waking state is tightly coupled to baseline neuronal activity [20, 62, 69]. Anaesthetized and sleeping mice also manifest neurovascular coupling, but this is overshadowed by dominating 0.04 Hz vasomotor waves [70]. Human fMRI data, including MREG data, corroborate the eclipsing during sleep or under anesthesia of CBF fluctuations by physiological pulsations, including 0.03‐ to 0.05 Hz vasomotor waves [58, 71, 72, 73].

Human fMRI studies have revealed increased very‐low‐frequency BOLD fluctuations in posterior brain regions during light N1–N2 sleep and episodes of low waking vigilance [72, 74, 75, 76]. Deactivation cycles of the cholinergic nucleus basalis of Meynert neurons precede the widely distributed hemodynamic BOLD signal increases in posterior brain regions during sleep [73]. Brain electrophysiological K‐complexes detected in EEG are formed in the basal ganglia and lead to increased vasomotor tone extending over the peripheral vasomotor waves in the sleeping brain, which mask the underlying neurovascular activity [46, 77, 78]. The CSF pulsations are strongly coupled to the vasomotor waves and K‐complexes, but their causal relationship or sequence remains uncertain.

Brain solute transport and water convection increase markedly during sleep [6, 40, 42]. The increased hydrodynamics occurring in sleep reduce the local intraparenchymal electrolyte concentrations, which reduces neuronal activity from awake fast rhythms to classical sleep delta power [42, 79]. In human MREG data, the brain areas of increased slow wave (delta) activity present marked increases in all three physiological brain pulsations, with strong increases in the low‐frequency vasomotor and respiratory pulsations, but lesser increases in the higher frequency cardiovascular pulsations [25, 80]. Sleep stage and rebound sleep after sleep deprivation also increase the power of vasomotor and respiratory pulsations [80].

**FIGURE 4:**
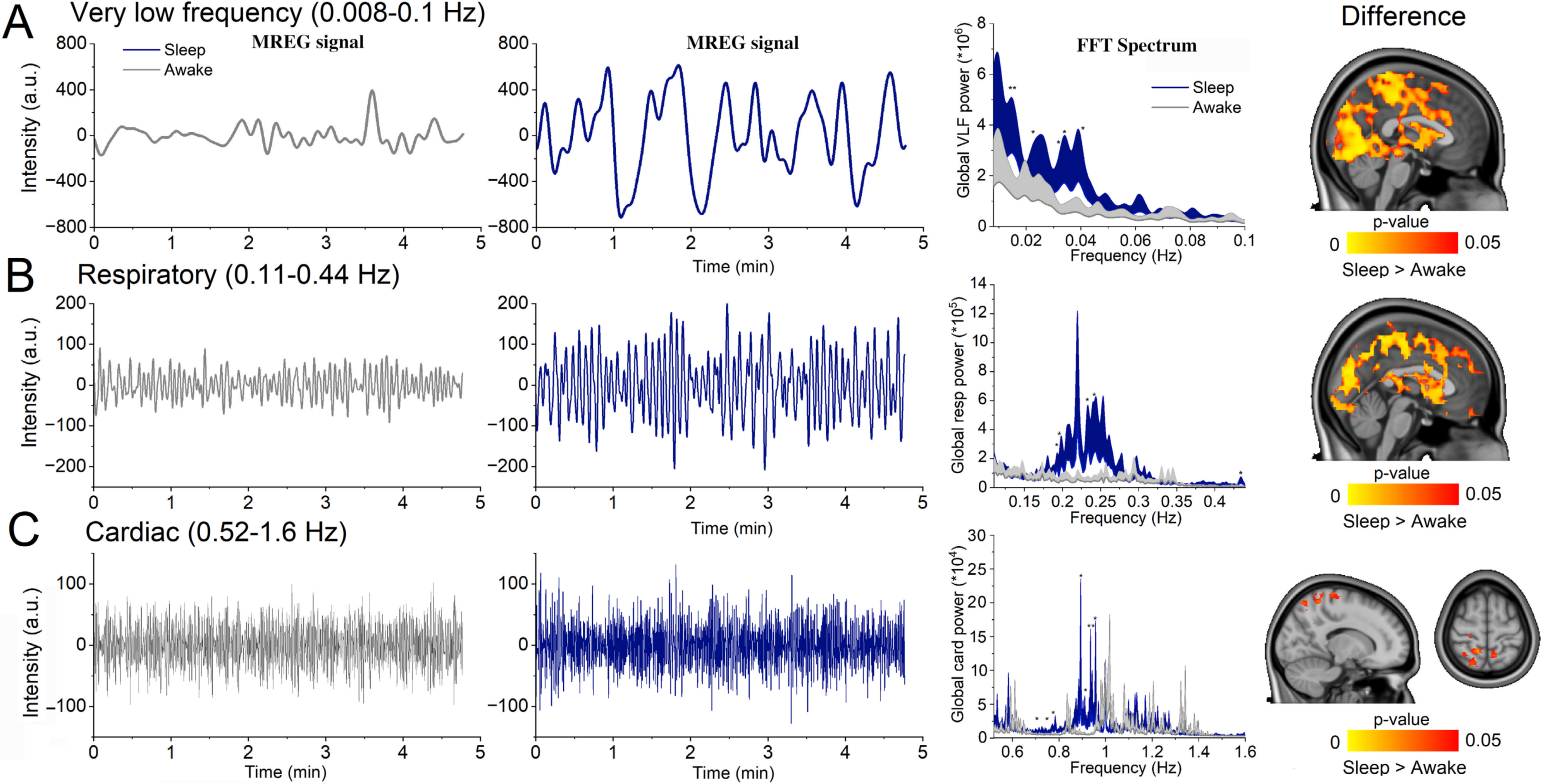
Physiological brain pulsations increase during sleep. (A) Very low‐frequency (0.008–0.1 Hz) pulsations during waking and NREM sleep (left) and their FFT power spectrum (middle) (*p* < 0.05). (B) Respiratory pulsations (0.11–0.44 Hz) during waking and NREM sleep (left), their FFT power spectrum (middle), and map of statistically significant differences (*p* < 0.05). (C) Cardiac (0.52–1.6 Hz) pulsations during waking and NREM sleep (left), their FFT power spectrum (middle), and map of statistically significant differences (*p* < 0.05). All pulsations increased during sleep versus waking state. Figure integrated from [81].

Vasomotor waves increase in amplitude during NREM sleep, cf. Figure 4A, which helps to drive injected CSF tracers along the perivascular space, in a manner resembling the intramural periarterial drainage (IPAD) mechanism where solutes are moved by the pumping action of the smooth muscle cells [32, 33, 34]. The altering frequency distributions over all the three physiological pulsation frequencies introduce a net reduction in the spectral entropy of EEG [82] and likewise the critically fast MREG signals [80, 81]. The ultrafast MREG signal can itself serve to separate sleep from awake epochs with nearly 90% accuracy according to ROC AUC analysis [81].

Upper‐airway resistance increased venous/CSF pulsations due to increases during sleep [83, 84], which may bear a relation to the occurrence of increased respiratory frequency power seen in the brain MREG signal [80, 81]. The supine position in sleep redirects venous outflow to the jugular vein, away from the paraspinal venous plexus drainage that predominates in the prone position; this shunting may further influence the respiratory brain pulsatility [85]. Breathing rate is less variable during sleep [83], which partially explains the reduced respiratory entropy in sleep [80, 81]. The concomitantly increased and stabilized vasomotor and respiratory pulsations, Figure 4A,B, respectively, in sleep would likely increase CSF flow toward the neuropil, potentially thereby inflating the extracellular space, as occurs during sleep/wake transitions [42].

In our 2024 MREG study, the transition to sleep was accompanied by increased cardiovascular pulse power in the brain (Figure 4C) and reductions in the periphery, with reduced coherence between brain and periphery. Vasomotor waves, on the other hand, became significantly more powerful and synchronized between the periphery and the sleeping brain. Most interestingly, there was an increase during sleep in the synchrony between the arterial vasomotor waves (CHE) versus venous BOLD waves in areas close to the parasagittal efflux areas, suggesting a more direct hydrodynamic effect via the interstitium and/or their associated blood capillaries. In these same brain areas, altered slow wave delta EEG power indicates areas of increased ISF/CSF interaction [80, 81, 86]. Recent studies in 2025 indicate that in these areas, the NREM sleep increases the propagation speed of vasomotor waves [87] and the waves start to regionally drive baseline DC‐EEG electrical potential of the brain as well as cortical hydrodynamics, suggesting increased tissue water convection over glia limitans [88]. These studies are virtually identical to invasive mouse studies where norepinephrine was shown to drive the vasomotor waves with opposing hydrodynamic waves [89].

Ultrafast MREG scanning opens a new viewpoint into several novel physiological phenomena; it can noninvasively detect very low‐frequency BOLD pulsations and individually separate them with a clear margin from respiratory and cardiovascular pulsations due to critical sampling of all neurofluidic signals, enabling precise analyses of each of them separately (Figure 4A–C). Moreover, it can detect how the strong respiratory pulsations modulate the amplitude of cardiovascular pulsations inside the brain (CREM). It further separates arterial vasodilations that precede slow BOLD responses in time, allowing estimations of causal delays between different physiological phenomena. Ultrafast scanning allows the detection of normal physiological changes such as arousal state and sleep‐related power and speed increases in CSF solute convection.

## 7 Clinical Applications

The critical MREG data sampling, in our opinion, offers a reasonable solution for the lack of clinically usable fMRI BOLD scanning. The key issue to the present lack of clinical applications of BOLD imaging has been the fact that slow BOLD scanning yields too noisy data that has too much variance between subjects for robust clinical scanning. In our experience spanning over a decade, the critically sampled MREG signal introduces noise reduction, and by separating the individual cardiorespiratory frequencies, it also enables accurate individual diagnostic imaging and mortality prediction against healthy reference population data [90, 91, 92, 93].

The initial investigations of the physiological brain pulsations reported fast Fourier transformation (FFT) power in the analysis of BOLD data [58, 94]. In a later approach, Yu‐Feng Zang calculated a square root of the FFT power, i.e., the amplitude of (very) low‐frequency BOLD signal fluctuations (ALFF) [95]. The BOLD_ALFF_ has since then become an important and robust [96] metric for detecting brain pathology, proving to be generally successful in detecting disease‐related changes in several meta‐analyses performed of several major neuropsychiatric disorders based on the > 1480 articles so far appearing in PubMed by the use of ALFF as a search keyword.

However, given the high variance in the reported ALFF findings in the literature, the similar power of vasomotor waves and respiratory pulsations normally prevailing in the cerebral cortex may well be due to a confounding factor from aliased respiration over ALFF, since virtually all studies have hitherto sampled the data noncritically with respect to faster cardiorespiratory pulsations [4, 97]. As described above, MREG avoids the aliasing and—based on our experience with > 2500 MREG scans—this precision of the method essentially avoids signal aliasing, such that other modulations (i.e., CHE and CREM) can be assessed separately [4, 25].

## 8 Alzheimer's Disease (AD)

**FIGURE 5:**
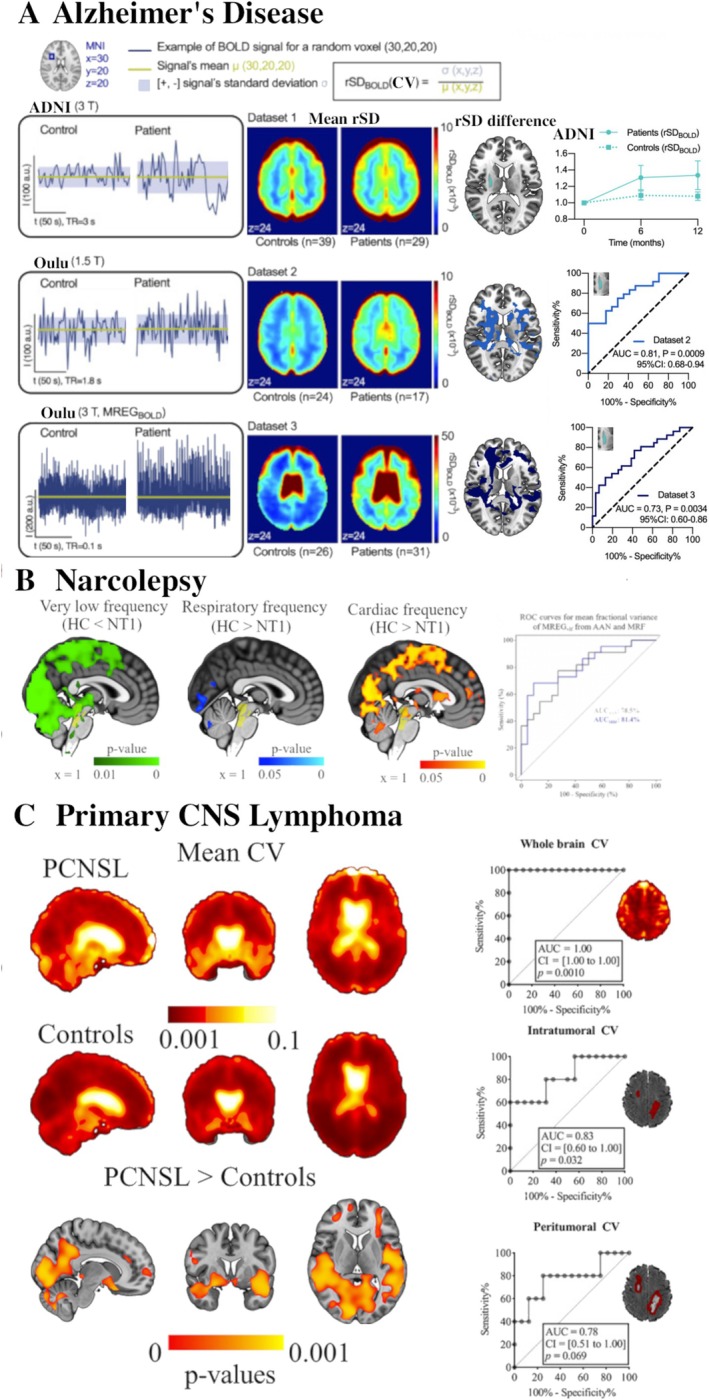
Physiological brain pulsations in different brain diseases. (A) Examples of BOLD signal coefficient of variation (CV, a.k.a. relative standard deviation [rSD] over the mean) and respective CVmaps of three independent Alzheimer's disease datasets: adNI 3 T EPI, Oulu 1.5 T EPI, and 3 T MREG data for representative control and ad time signals. Group mean CV maps on the right indicate the respective increases in BOLD signal CV for the AD patients in all datasets, even those with different field strengths and TRs in the range 100–3000 ms. ADNI data showed consistent elevation in AD CV predating cognitive decline, not detected in matched controls, in repeated measures at 6 and 12 months [104]. ROC curves indicated good diagnostic accuracy of BOLD CV in AD, adapted from [104]. (B) Narcolepsy patients (NT1) showed increased MREG signal variance in VFL frequencies, < 0.1 Hz, but decreased in respiratory and cardiovascular frequencies. The MREG signal from AAN and MRF nuclei enabled good separation between controls and narcolepsy subjects, modified from [91]. (C) The presence of primary CNS lymphoma growing along perivascular routes of an intact BBB increased MREG CV to an extent exceeding the pathology as indicated by Flair or T1contrast MRI. Thus, whole‐brain CV analysis is markedly more specific and sensitive than intratumoral and peritumoral CV analyses. A whole‐brain analysis CV quantifies the general spread of pathology, and offers the only existing precise prediction from pre‐treatment data of any kind of clinical information on primary CNS lymphoma (PCNSL) mortality, despite 80 % curative i.a. BBB opening augmented chemotherapy adopted from [93].

Disturbance of the glymphatic clearance pathway that is driven by the physiological brain pulsations may be a contributing factor to the amyloid/tau aggregations typical of ad. Moreover, soluble Ab added to CSF jams the solute convection on its own [98]. Much as with ALFF, the BOLD_CV_ (std/mean) shows alterations in ad, FTD, and likewise in small vessel vasculopathies [99, 100, 101, 102, 103]. Indeed, the physiological brain pulsations influence the variation of the BOLD signal. Using three independent datasets, including local 1.5 and 3 T adNI functional BOLD scan data and 3 T MREG data, we showed that BOLD_CV_ is indeed increased in ad patients (Figure 5A). The BOLD_CV_ offers predictive scanning as it increases permanently in ad patients 6 months prior to the measured cognitive decline in the adNI dataset. The ultrafast MREG BOLD_CV_ method captured the CV results also from 1.5 and 3 T data obtained by a slower scanning protocol, i.e., TR 1800 and 3000 ms versus 100 ms in MREG. Our analysis of the MREG data enabled a closer examination of the separate physiological sources and indicated that the greatest perturbation of MR signal variance in ad patients occurred in the cardiovascular frequency, within ad, and also somewhat in the respiratory band, and not so much in the VLF [104]. Furthermore, BOLD_CV_ seems to be added into clinical diagnostics as it can differentiate ad, FTD, and schizophrenia with good accuracy [103].

**FIGURE 6:**
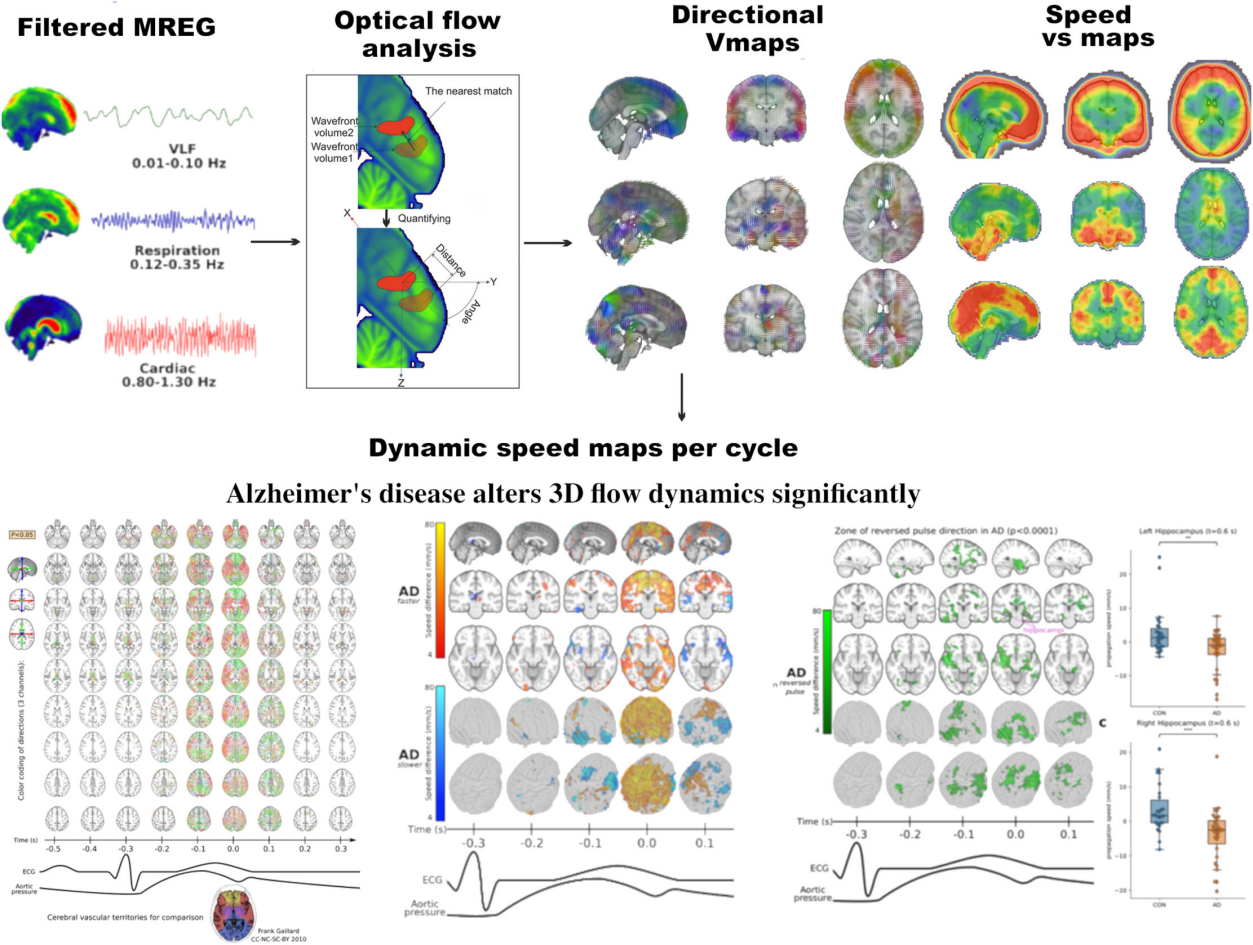
Measuring the velocity of brain pulsations with MREG optical flow (OF) analysis. The preprocessed MREG data are first bandpass‐filtered into the subject's own physiological pulsation time‐domain signals. The optical flow algorithm is applied to detect motion in the 3D time domain data, either by following MREG signal features like the cardiac pulse nadir (sparse OF) or the composite of all propagating signal features (dense OF). Optical flow of MREG data enables quantitation of 3D features of movement: the velocity with direction (*V*) and magnitude of speed (*v*_*s*_) in standard MNI space. Ultrafast sampling enables dynamic quantitation of propagation velocity as a function of the phase of each averaged physiological pulse, which helps to assign a physiological meaning to any detected pathology. In the ad brain (left), the most significant changes in cardiovascular impulse velocity occur along arterial territories. The cardiovascular impulses are somewhat slower near major artery branches but markedly faster (middle, hot color, *p* < 0.005) in the narrowed intraparenchymal arteries in ad during the arrival of the systolic arterial impulse. Importantly, the method detected new pulses that are reflected back in the opposite direction of their propagation (right panel, green color, *p* < 0.05) from these narrowed parenchymal arteries [66, 105], cf. dynamic 3D videos of pathological pulsations in the original article and Supporting Information.

The ultrafast MREG scanning protocol also enables 3D quantitative assessment of the effects of physiological brain pulsations in SI units, such as the velocity of pulse propagation inside the brain and CSF spaces; see also Figure 6. Optical flow analysis, which is a standard tool for assessing spatial propagation of video image features, supports the analysis of abnormal cardiovascular impulses in ad brain [66, 105], cf. Figure 6. The optical flow images in such patients revealed increased velocity of cardiovascular impulses in the peripheral arteries, and an unexpected *reversed* cardiovascular impulse propagation in (para)hippocampal areas of ad patients reflected back from narrowed intraparenchymal arteries [105] (Figure 6). Recent research results indicate disruption of BBB integrity in these very brain structures, irrespective of the extent of amyloid/tau aggregations [106]. Importantly, the perivascular Virchow–Robin spaces between the astrocytic endfeet (glia limitans) and basement membranes/smooth muscle cells are structural components of the BBB, consistent with a link between failure of the vascular pulsations that normally drive the convection of solutes within the BBB [107].

Pathological mechanisms can alter the physical brain pulsations driving blood and CSF flow at different time scales. Changes may be sudden, such as the cessation of blood/CSF flow upon cerebral infarct, which results in failure of mitochondrial metabolism in a matter of seconds, whereupon brain edema due to spillover of perivascular CSF water into the interstitium follows [6, 108]. An acute brain trauma can affect a surge of cerebral noradrenaline release, which promptly arrests VLF pulsation and glymphatic clearance; in an experimental model of traumatic brain injury (TBI), treatment with a pan‐adrenergic cocktail of alpha and beta adrenergic receptor antagonists is conducive to long‐term recovery [109].

Other pathophysiological changes can arise over decades. For example, mean arterial blood pressure often increases with age, as the progressive loss of elastin and collagen stiffens arteries, which then reduces cardiovascular pulsatility [110] and increases in BBB permeability [106]. Hypertension is a risk factor for both ad and stroke, as it reduces vessel‐wall pulsatility and perivascular CSF convection [7, 110]. Also, the polar periarterial astrocytic expression of AQP4 water channels declines with age, which may promote the accumulation of amyloid in perivascular structures, ultimately leading to cognitive impairment in the context of ad [ 111, 112].

## 9 Narcolepsy

Narcolepsy Type 1 (NT1) is a sleep‐related neurological disorder characterized by abrupt changes in arousal, including sleep attacks, cataplexy, and fragmented nighttime sleep, which arises because of decreased signaling by the receptors of sleep‐regulating hormone orexin (a.k.a. hypocretin) in the hypothalamus. As glymphatic brain clearance normally increases during sleep, the sleep dysregulation in patients with narcolepsy presents a target for investigating the relationship between sleep patterns and physiological brain pulsations driving glymphatic flow. Indeed, narcolepsy shows an association with AD in some patients, suggesting a causal relationship [113].

MREG imaging at the critical sampling rate of 100 ms enabled our investigation of information propagation between independent functional brain networks in a group of NT1 patients in comparison with healthy volunteers. The temporal lags between these networks revealed a monotonous and delayed propagation of internodal activation, particularly with respect to the interactions between the awareness‐related default mode network and other brain networks in NT1 patients [114].

A detailed analysis of the differing physiological brain pulsations between NT1 patients and controls revealed an interesting bidirectional change in all physiological pulsations [91]. Notably, vasomotor VLF variance was higher in the NT1 group, whereas cardiorespiratory variance was higher in the controls (Figure 5B). In clinical terms, an ROC analysis demonstrated that the pulsations enabled the distinguishing of patients and controls, whereas the amplitude of the cardiac‐related pulsations correlated with subjective disease severity (Figure 5B). Collectively, these findings suggest that functional connectivity is delayed in narcolepsy, along with opposing changes in the main drivers of intracranial hydrodynamics [91, 114]. Compared also with sleeping controls, the vasomotor waves have increased power in certain areas in awake narcoleptic subjects, underlining the importance of hyporecretin sleep hormone in the control of human brain pulsations [92].

## 10 Primary CNS Lymphoma

Primary CNS lymphoma (PCNSL) is one of the most aggressive types of intracranial tumors, where pathological lymphocytes infiltrate the brain by passing along perivascular spaces. This invasion markedly changes (peri)vascular structure and hydrodynamics. In our hands, the physiological variation depicted by MREG signal CV was significantly increased in PCNSL patients compared with controls (Figure 5C) [93]. After adjusting for medication status, head motion, and age, the patients revealed higher CV values (group median 0.035) than healthy controls (group median 0.024) around arterial territories (*p* ≤ 0.001). Comparison between CV clusters and findings from gold standard clinical neuroimaging (i.e., contrast‐enhanced, diffusion‐weighted, fluid‐attenuated inversion recovery [FLAIR] data) indicated that the CV increases exceed the pathology seen on the more conventional clinical scans [93]. Abnormal clusters (median 1.10 × 10^5^ mm^3^) extended spatially beyond the FLAIR lesions (median 0.62 × 10^5^ mm^3^), with significantly higher volumes (*p* = 0.0055).

Importantly, as a general measure of disease involvement, the whole‐brain mean CV predicted short‐term mortality with 100% sensitivity and 100% specificity, as all deceased patients who died during follow‐up had shown significantly higher mean CV values (*n* = 5, median 0.055) than did the surviving patients (*n* = 16, median 0.028) (*p* < 0.0001), cf. Figure 5C) [93]. This underlines the accuracy of the MREG scanning, as it offers a new, precisely predictive clinical tool that otherwise does not exist in any other diagnostic modality.

Based on these observations, we argue that the spatial extent of lymphocyte infiltration can be detected from its effects on physiological brain pulsations, with a sensitivity exceeding that available from current clinical detection methods. The MREG procedure opens a new channel for predictive scanning biometrics quantified based on the detection of the global extent of lymphocyte infiltration through its pathological effects on brain hydrodynamics. This promising new field of physiological scanning has direct clinical implications and value.

## 11 Epilepsy

Intractable epilepsy can entail the ablation of normal AQP4 water channel expression in the astrocytic endfeet lining the perivascular space [115]. The disposition of AQP4 channels bears a relation to disrupted water movement, through consequent perturbations in osmotic gradients between the neuropil and perivascular space [116]. Intracranial EEG measurements in intractable epilepsy patients show a strong association between electrophysiological abnormalities with respiratory brain pulsations [117, 118].

In a series of clinical studies, we utilized MREG to obtain crucial insights into the relationship between brain pulsations and the pathophysiology of focal epilepsy [119]. Indeed, repeated scanning reveals perturbation in all three physiological pulsations, most distinctly in the respiratory brain pulsations of focal epilepsy patients [120].

**FIGURE 7:**
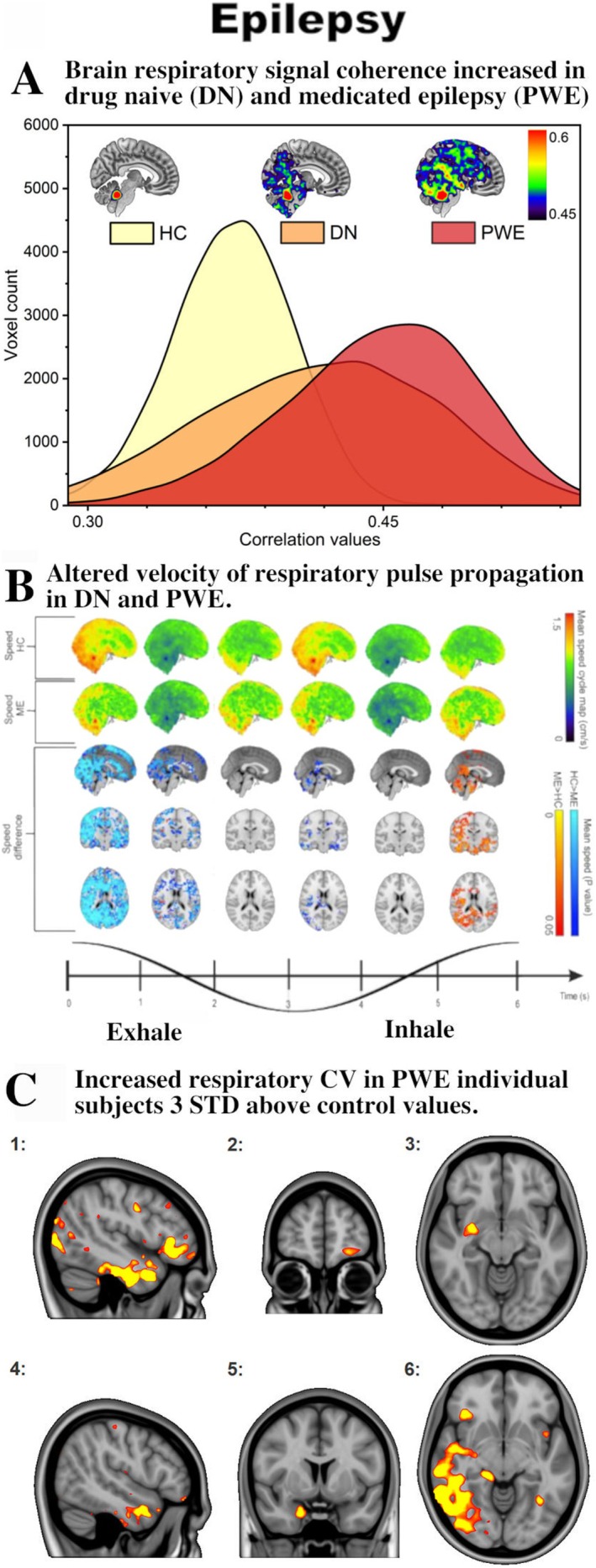
Physiological brain pulsations in epilepsy. (A) Respiratory pulse coherence with whole‐brain MREG signal increases in drug‐naïve and especially in medicated patients with epilepsy, in comparison with matched healthy controls, modified from [121]. (B) In focal epilepsy, the respiratory pulsation velocity decreases (light blue color, *p* < 0.05) in early exhalation and slightly increases at the end of inhalation, indicating altered dynamics of the respiratory‐driven brain hydrodynamics (hot color, *p* < 0.05) [122], cf. dynamic videos in the Supporting Information). (C) Examples of individual detection of increased regional respiratory FFT power in six patients with epilepsy at 10 STD above the values in age‐matched healthy controls. Modified from [90], cf. 3D videos of dynamic speed changes over the respiratory impulse cycle in drug naïve and chronic epilepsy patients in the original article.

The epileptic brain shows greater synchronization of respiratory impulses (Figure 7A) [121]. This synchronization correlated positively with seizure frequency, thus demonstrating promising diagnostic accuracy for distinguishing patients from controls. Finally, measurements of propagation velocities revealed complex dynamics in the changes of propagation speeds of the respiratory brain pulsations, which slowed down during early exhalation and increased in late inhalation in comparison with healthy control optical flow patterns (Figure 7A,B) [122].

Importantly, ultrafast MREG sampling now makes possible individual diagnostic mapping of pathology in seizure patients through recording of respiratory brain signal variance normalized to age‐ and gender‐matched healthy control data. We were thus able to localize individual abnormalities > 3 STD above normal variance values in up to 80% of medicated patients with epilepsy and drug‐naïve seizure patients (Figure 7C) [119, 120]. The CV abnormalities are matched with the severity of clinical symptoms from patient records [120].

In contrast, conventional anatomical MRI can usually reveal pathology in only 10% of de novo seizure patients, and slowly sampled conventional fMRI with signal aliasing performs little better. Combined EEG and fMRI recordings are sometimes helpful, but with limitations due to the generally low incidence of seizures while in the scanner [119]. The high sensitivity of MREG to abnormal baseline brain physiology indicates a more direct detection of an underlying hydrodynamic pathology that makes the brain vulnerable to seizure activity. The respiratory wave driving of brain hydrodynamics seems to be altered in focal epilepsy, which implies failure in (peri)venous flow that may reflect failure of venous return as well as glymphatic solute efflux. We suppose that perturbation in these key homeostatic mechanisms might promote epileptic activity by altering interstitial electrolyte and transmitter concentrations.

Because of the critical sampling rate, ultrafast MREG offers precise signal noise control and removal of interindividual data variance that stems from individual cardiorespiratory rates. In our 12‐year experience, MREG turns fMRI finally into a very feasible diagnostic tool when one utilizes the individual physiological pulsations as biomarkers against normative data; it enables individual‐level diagnostics at good to excellent accuracy (ROC AUC 0.7–0.9). Importantly, MREG offers the only existing diagnostic tool for predicting mortality for PCNSL from data taken before curative BBBD therapy. In our experience, MREG is a valid diagnostic tool due to focusing on individual physiological pulsations related to brain solute transport.

## 12 Technical Considerations

The MREG technique is currently available for 3 T Siemens scanners (Prisma, Vida) XA30 & XA 60 and older Skyra versions. However, plans are underway to obtain broader implementation in other vendors' MR scanner types, namely, the work of Prof. Maxim Zaitsev in Freiburg University toward developing a new Pulseq multivendor sequence coding tool in Matlab. Because of technical issues, MREG does not currently run on 7 T scanners but should be transferable to the setting of 1.5 T, thus making the technique potentially available at most hospital centers.

The high 100‐ms 3D temporal resolution required by MREG entails undersampling of the k‐space by a factor of 18 (2.6 per direction). The MREG sequence gathers k‐space in a single‐shot stack‐of‐spirals (SOS) in 80 ms, with alternating counterclockwise/clockwise spirals that reduce off‐resonance artifacts. Compared with classical BOLD sequences, the ultrafast SOS undersampling of k‐space brings a penalty regarding lesser sensitivity to field inhomogeneities and a broader point spread function at the center of the field of view (i.e., deep brain structures) compared with the cerebral cortex. However, because of the concentric design with a lack of multiple self‐crossings of the k‐space center, MREG also minimizes the pronounced signal loss in areas with strong susceptibility effects and field inhomogeneities as compared with other ultrafast sequences.

MREG imaging is accomplished using parallel imaging, which is currently applicable using head coils with 20, 32 to 64 channels, although extension to higher channel numbers is a work in progress. The parallel reconstruction tends to show artifacts arising from the high undersampling factor, thus calling for a regularized reconstruction method. As pointed out by the original developer of MREG technology, “Nominal spatial resolution is 3 mm isotropic, but the actual resolution is lower and anisotropic due to the nature of iterative reconstruction of the highly undersampled acquisition. For reference a standard double echo gradient echo sequence are performed prior to the MREG‐acquisition, which is used to calculate coil sensitivity profiles as well as a 3D field map” [29].

An important practical issue for the scanning of physiological signals is the spoiler/crusher gradient, which controls the spin history effects from the flowing saturated spins that are always present in 3D acquisitions. Importantly, if the crusher is omitted, there emerges a problematic stimulated echo that renders the signal useless. On the other hand, for whole‐brain imaging of the physiological pulsations driving CSF and its solutes, the spin history effects are not merely a nuisance, but convey important information about the spin of flowing water. Thus, we have optimized the MREG method for a crusher gradient value close to the minimal useful value of only 0.1 (10%). Applying the full value of unity (100%) would minimize the flow effects at the expense of increasing the relative influence of susceptibility effects. The crusher is applied during the remaining 20 ms to fulfill the round 100‐ms TR.

The flip angle is another sequence parameter having clear effects on the pulsation signal. As elegantly shown by the group of Jerzy Bodurka, a low flip angle can actually confer improved sensitivity to functional information, despite the inherently lower signal amplitude [123]. We have observed a similar sensitivity of the pulsations to the flip angle, which leads us to favor scanning at both flip angles of 5° and 25°, the latter of which slightly exceeds the grey matter Ernst angle of 23°. The flip angle deserves deeper analysis, as it can significantly affect the sensitivity of the MREG data to different physiological aspects. There is now work in progress to unravel the effects of FA on the MREG data.

The TR could in theory also be shortened from 100 ms to as tight as 80 ms, which would increase the critical, nonaliased Nyquist sampling range of the MREG signal from 5 to 6.25 Hz. The k‐space center is always attained in an echo time (TE) of 33 ms. As there are inevitably other issues arising in relation to changing such parameters, we suppose that systematic investigations of MREG signal and flow effects with respect to crusher strength, FA, and TR will soon yield some clarification of the issue. For a more thorough review on the MREG sequence, we refer the reader to Jurgen Henning's recent article “15 years of MR‐encephalography” [29].

Currently a significant limitation of MREG lies in its use of iterative back‐reconstruction instead of the usual FFT reconstruction per 2D slice used in conventional EPI, which is necessitated by the highly undersampled k‐space coverage of the MREG SOS k‐space gathering [29, 124]. This procedure is performed off‐line in a Linux environment with a Matlab code provided along with the sequence. The dynamic off‐resonance correction of k‐space (DORK) incorporated in the reconstruction significantly further reduces the off‐resonance artifacts due to B_0_‐field shim changes induced by respiratory movements of the thorax and abdomen [124]. The iterative reconstruction as performed with Tikhonov regularization of the L1‐norm produces sharper but noisier images. The computation currently entails some 2–3 min per computer CPU per 3D brain volume. Given the requirement of 3000 brain volumes for each 5 min of data recording for adequately sampling slow vasomotor waves, image reconstruction is now the rate‐limiting process; a single 5‐min MREG sequence thus requires nearly 1 week to reconstruct with a single CPU laptop.

The approach of iterative reconstruction can be allocated to parallel C/GPUs processing. Parallel net‐computing clusters with hundreds or thousands of C/GPU processing units are becoming increasingly available, which can obtain adequate image reconstruction of the MREG data. Our OFNI group has scanned and reconned more than 2500 brain volumes since 2012 by parallel processing with a few hundred C/GPUs, both locally and at the national CSC computing facility. To date, the AI‐based machine learning methods have not sufficed for detecting the subtle image‐to‐image changes that report brain pulsations. The back‐reconstruction problem is ill‐posed to current AI algorithms; future innovations in AI algorithms and other computing strategies may pave the way for faster reconstruction without loss of the intricate physiological pulsation signals. Also, recent development in GPU processing speeds seems to offer finally a fair solution for the image data reconstruction.

The spirally acquired MREG signal is spatially very precise, but images are somewhat blurry, and the 3 × 3 × 3 mm spatial accuracy still has a moderate point‐spread function. In anatomic terms, each 27‐mm^3^ voxel does contain signal partial contributions from CSF, ISF, arteries, and veins, and is further subject to partial volume effects, especially at the margins of the human brain. If there is a need for markedly higher spatial resolution, one can always use other, more standard MRI sequences during the same scanning session. But greater anatomical precision, as well as diffusion weighting, phase encoding, not to talk about more complex MR spin manipulations, invariantly takes time, which one does not have if one wants to avoid the aliased influences of individual physiological pulsations.

However, as stated in the Clinical Application, the key thing that has previously prevented fMRI from becoming a clinically valid tool has been thought to be its “inherent noisiness.” This assumed noise actually stems from the slow sampling of data that prevents detection of *individual* cardiorespiratory pulsation frequencies, which furthermore *individually* aliase over slow vasomotor BOLD changes, rendering the data apparently very noisy also between subjects. The fact that we all breathe and our hearts beat differently has been previously completely overlooked by slow fMRI. But when actually using each individual's own cardiorespiratory rates, suddenly the high statistical power in data like MREG turns into a clinically precise tool offering individual‐level diagnostics against a normative population data and can even offer the only tool that gives 100% accurate mortality prediction of pretreatment data, as shown earlier in this review.

This all implies that the major avoidable source of the intersubject fMRI noise has been the noncritical sampling of data. The previously neglected critical sampling can now be avoided by MREG or by other such sequences as VEPI, INI, and GIN, where the TR is clearly < 200 ms. If one wants to scan anatomically more accurate data, one option is to have to low TR but scan high anatomical precision with commonly available T2*‐weighted sequences in a limited number of slices, usually 1–2. Limiting spatial coverage to yield high spatial precision information on key structures has been used in techniques such as in fMREye, where intraorbital pulsations driving eye and perineural CSF flew around the optic nerve have been successfully detected [125]. In the future, we hope to see scanners with more advanced coils that can also improve the spatial accuracy further, while still maintaining a critical sampling frequency of at least 10 Hz.

## 13 Signal Analysis

Based on our experience, the previous fMRI data preprocessing and analysis tools are quite valid and usable for MREG data [29]. Our group has tested preprocessing using the FSL melodic pipeline and the AFNI tools detrending, despiking, highpass, and motion control. However, for MRED data, linear registration offers more accurate spatial registration based on individual T1w 3D mprage images into MNI152/305 or HCP standard space than nonlinear, which tends to spatially warp the data somewhat.

For physiological signal power analysis, a simple FFT power or amplitude projections into MNI space can be used to provide spatial maps of each pulsation strength, cf. Figure 1 [58]. AFNI 3DTproject and 3DFFT perform robustly even with thousands of 3D brain volumes. The amplitude of the low‐frequency fluctuations (ALFF) and faster pulsations is calculated by simply taking a square root of the FFT power maps [95]. There are several reports indicating an exact match between cardiorespiratory signals measured from the brain match with peripheral physiological recordings [104, 114, 121].

As the 10‐Hz MREG sampling is critically fast, the measured signals are practically devoid of physical signal aliasing. Consequently, simply applying a bandpass filter to the data can edit out or enable an analysis of the physiological signals directly from the BOLD recordings (Figures 1 and 4). The simple FFT bandpass approach avoids the risk of misleading data distortions that are introduced by global signal regression. Importantly, as the physiological pulsations move across the entire brain cycle after cycle, each voxel experiences a somewhat different phase in each pulsating signal. Thus, an averaged time domain signal regression, even with dozens of regressors, cannot completely accommodate the moving physiological noise, since the regressors are virtually always in the wrong phase compared with the detected signals per given voxel, no matter where or how they are derived. Therefore, only FFT‐based noise handling serves for physiological signal removal, as precisely pointed out by Chen and co‐workers [80].

The physiological pulsations were long regarded merely as noise masking activation responses in fMRI. As argued throughout the review, the ultrafast scanning of pulsations in fact offers novel functional contrasts for detecting faster responses to neuronal activity via measuring arterial vasodilatations with CHE. This results in more accurate connectivity lag measurements between functional networks [126, 127, 128], while also supporting the quantitation of hydrodynamic pulsations that drive the convective clearance of brain metabolic wastes from the brain (Figure 2).

As the MREG protocol gives nonaliased BOLD signals in the VLF range, it is compatible with all previous functional activation and resting state analytical approaches, with the added benefit of significantly increasing accuracy due to both increased statistical power and lack of aliasing [29]. The BOLD pulsations in the VLF domain can be imaged with good repeatability as compared with classical EPI results [15, 29].

In the physiological domain, pulsations can now be analyzed in detail, extending beyond simple considerations of pulsation amplitude power to enable a visualization of the modulations between the several pulsations that would be impossible in the presence of aliasing [4]. The coherence or correlation of the pulsations can be measured across the entire brain, as in the example of whole‐brain correlations with respiratory pulsations measured in the fourth ventricle [46, 73]. The signal coefficient of variation (a.k.a. the standard deviation relative to the mean) of the pulsation signals is a highly sensitive biomarker in several patho (physio)logical conditions [74, 106, 119]. Spectral entropy, which designates the information content of the MREG signal frequency spectrum, is also a promising biomarker, not least of all in relation to sleep‐related changes in brain activity [91, 92] (see section Sleep). These metrics are available from the MREG maps through simple calculation by fMRI tools and other signal analysis such as R and Matlab.

The optical flow analysis of speed is an established way of measuring fluid movement, which now serves for quantifying to propagation speed of physiological pulses passing through the brain, cf. Figures 6 and 7 and Supporting Information [66]. Optical flow introduces 4D results, as it depicts flow velocities in 3D orientations as a function of the wave cycle phase. This introduces a novel feature of fMRI of the brain, in that pulsation signals are now quantifiable in SI units (m/s), with potential generalization for their first derivatives [66, 105, 122]. It is possible to depict and analyze such results dynamically and without triggering as functions of the cardiorespiratory and vasomotor cycles. Although power and velocity of brain waves are readily graspable concepts, they can convey a wide variety of new information pertaining to the physiological variability, risk factors for or prodromal detection of neurological disease, or end‐stage pathological conditions.

The rapid MREG scanning increases the precision of measurements in a simple relation to the greater volume of data sampled per second, which substantially increases the statistical sensitivity of the measurements. Circumvention of physiological signal aliasing is a vital aspect for eventual application of the technique in individual‐level diagnostics based on direct observation of pathophysiological mechanisms of disease and their mapping in the common space, i.e., MNI152/305. For comparison with individual cases, we have in the past decade assembled a normative database comprising hundreds of individual cases. Looking ahead, we foresee that a more extensive normative data set extending across all ages should enable the detection of individual deviations from the norm, as would be required for diagnostic applications. Such a diagnostic approach would represent a noninvasive mapping of subtle changes in brain fluid dynamics that are becoming increasingly important in the literature as early forms of pathology predating neuronal dysfunction.

Technically, MREG is a T2*‐weighted ultrafast spiral sequence for collecting whole‐brain MRI data at a staggering 10 Hz rate (i.e., TR 100 ms) by undersampling k‐space with a center focus. MREG maximizes time resolution with a minimal penalty on spatial accuracy. In addition to the classical susceptibility‐weighted BOLD contrast, the MREG also offers phase contrast for simultaneous mapping of the fast arterial impulse dynamics. The current technical limitation has been image reconstruction that requires considerable parallel processing due to the iterative back‐reconstruction. However, this technical limitation can be solved with recent advances in parallel computing.

## Supplementary Information

Ultrafast MREG scanning offers novel insights into basic brain physiology and enables more direct observation of underlying neuropathology with markedly improved sensitivity compared with conventional imaging. We foresee that broader use of ultrafast scanning will enable more precise targeting and monitoring of future therapies for various brain pathologies.

## Supporting information


**Video S1** Video illustrating a mean cardiovascular brain signal impulse in three sagittal, axial, and coronal planes and a rotating 3D glass brain rendered into MNI152 space averaged from individually band‐passed recordings extending over > 15,000 cardiac cycles of 50 subjects. The video images represent demeaned *z*‐scored mean signal intensity with (red‐hot) positive 2–0 colors and light blue negative 0 to −2 *z*‐scores. Individual cardiac cycles are averaged with *circshift* into a 0.9‐s heartbeat, i.e., 67 bpm heartbeat [4, 85, 119]. FFT power in Figures 1 and 4 illustrates an individual example of a band‐passed power peak. Other examples of the cardiovascular brain pulsation signals are illustrated in Figures 1, 2, and 4. Here, the impulse was timed to its arrival at the anterior cerebral artery in front of the corpus callosum. For greater interpretability, the video plays at 10% speed. The negative signal drop (cold blue color) stemming from cardiovascular impulse arrival is discernible in the cerebral arteries some 0.3 s after heart systole. This signal drop originates from a phase/SSFP change in accelerating (peri)vascular water proton spins. The signals in CSF ventricles, the cerebral aqueducts, and the sagittal sinus show a compensatory inverse signal increase (hot color) during the systolic inflow of approximately 7–10 mL of arterial blood, as dictated by the Monro–Kellie doctrine. During diastole, these waves invert to show opposite signal intensity changes in these regions.

**Video S2** Video illustrating amplitude modulation of cardiovascular pulsations by respiration (cardiorespiratory envelope modulation; CREM) passing as a wave through the human brain [4]. Figure 1 in the article illustrates the heterodyne CREM power peaks on both sides of the cardiac peak, spaced at a distance exactly equal to the respiratory frequency on either side of the principal cardiac pulsation peak. The CREM signal is produced by band‐passing the MREG signal in the range of the cardiac 1‐Hz power peak and including both heterodyne peaks 0.2 Hz away from the cardiac peak, i.e., 0.8‐ to 1.2‐Hz‐wide bandpass. Then, an envelope is placed over the band‐passed cardiac signals from the same 50 healthy subjects appearing in Videos [Link], [Link], [Link], [Link]. The propagation of an averaged and phase‐synchronized 6‐s envelope cycle is then portrayed in MNI152 space in sagittal, coronal, and axial views along with 3D glass brain images. The video color encoding has the same positive and negative [2 to −2] *z*‐score thresholding as all Videos [Link], [Link], [Link], [Link].

**Video S3** Video illustrating the averaged respiratory band‐passed brain wave signal from the control 50 subjects, with phase‐correction into the same frequency. Individual respiratory cycles are averaged to *circshift* to a 6‐s length, and somewhat the movement in the simultaneous 3D glass brain slows the video down for better illustration in the 3D MNI152 standard space [4]. The timing of the pulse was obtained from the IV‐ventricle, which is most sensitive to the respiratory brain pulsations. A transient increase in the MREG BOLD signal intensity is driven by the respiratory pulsations that enter the posterior fossa CSF and venous structures. The respiratory wave then continues forward into the brain white and grey matter toward frontal regions, finally gathering around and into the parasagittal space, as described by Eide and Ringstad [12]. Inhalation both draws blood from the brain and also induces a countering CSF inflow toward the brain. This creates BOLD signal changes in veins and T2 flow signal changes in the CSF spaces. The video color encoding has the same positive and negative [2 to −2] *z*‐score thresholding as all Videos [Link], [Link], [Link], [Link].

**Video S4** Video illustrating the propagation of the very low‐frequency vasomotor (< 0.1 Hz) waves of 50 healthy subjects averaged in the same signal phase [4, 85]. The posterior cingulate cortex node of the DMN was used as an ROI to trigger the timing of the vasomotor wave pulse. The 10‐Hz MREG signal reflects a classical vasomotor BOLD signal devoid of the cardiorespiratory aliasing effects that typically obscure classical BOLD scans with TR > 300 ms. Notably, the movement of the VLF signal tends to gather into the venous collecting system, and the countering CSF pulsation is less powerful as compared with the counter‐pulsations present in the cardiorespiratory pulsations seen in Videos [Link], [Link], [Link]. The video color encoding has the same positive and negative [2 to −2] *z*‐score thresholding as all Videos [Link], [Link], [Link], [Link].

## Data Availability

The sharing of the data used in the research reviewed in this article is a work in progress in relation to current GDPR legislation. Patient and control subject informed consent to collaborative data sharing has been obtained. Data can be shared upon reasonable collaborative request to the first author.

## References

[1] Sulla circolazione del sangue nel cervello dell'uomo: ricerche sfigmografiche/del Angelo Mosso. Wellcome Collection, accessed February 1, 2022, https://wellcomecollection.org/works/rjhn3gtu.

[2] C. S. Roy and C. S. Sherrington , “On the Regulation of the Blood‐Supply of the Brain,” Journal of Physiology 11, no. 1–2 (1890): 85–158.17. 10.1113/jphysiol.1890.sp000321 PMC1514242 16991945

[3] H. Berger , Lehre Von Der Blutzirkulation in Der Schadelhohle Des Menschen Namentlich Unter Dem Einfluss Von Medikamenten (1901).

[4] L. Raitamaa , N. Huotari , V. Korhonen , et al., “Spectral Analysis of Physiological Brain Pulsations Affecting the BOLD Signal,” Human Brain Mapping 42, no. 13 (2021): 4298–4313, 10.1002/hbm.25547. 34037278 PMC8356994

[5] K. Gouveia‐Freitas and A. J. Bastos‐Leite , “Perivascular Spaces and Brain Waste Clearance Systems: Relevance for Neurodegenerative and Cerebrovascular Pathology,” Neuroradiology 63, no. 10 (2021): 1581–1597, 10.1007/s00234-021-02718-7. 34019111 PMC8460534

[6] N. A. Jessen , A. S. F. Munk , I. Lundgaard , and M. Nedergaard , “The Glymphatic System: A Beginner's Guide,” Neurochemical Research 40, no. 12 (2015): 2583–2599, 10.1007/s11064-015-1581-6. 25947369 PMC4636982

[7] H. Mestre , J. Tithof , T. Du , et al., “Flow of Cerebrospinal Fluid Is Driven by Arterial Pulsations and Is Reduced in Hypertension,” Nature Communications 9, no. 1 (2018): 4878, 10.1038/s41467-018-07318-3. PMC6242982 30451853

[8] T. P. Santisakultarm , N. R. Cornelius , N. Nishimura , et al., “In Vivo Two‐Photon Excited Fluorescence Microscopy Reveals Cardiac‐ and Respiration‐Dependent Pulsatile Blood Flow in Cortical Blood Vessels in Mice,” American Journal of Physiology—Heart and Circulatory Physiology 302, no. 7 (2012): H1367–H1377, 10.1152/ajpheart.00417.2011. 22268102 PMC3330793

[9] V. Kiviniemi , X. Wang , V. Korhonen , et al., “Ultra‐Fast Magnetic Resonance Encephalography of Physiological Brain Activity–Glymphatic Pulsation Mechanisms?,” Journal of Cerebral Blood Flow & Metabolism 36, no. 6 (2016): 1033–1045, 10.1177/0271678X15622047. 26690495 PMC4908626

[10] S. Dreha‐Kulaczewski , A. A. Joseph , K. D. Merboldt , H. C. Ludwig , J. Gärtner , and J. Frahm , “Identification of the Upward Movement of Human CSF In Vivo and Its Relation to the Brain Venous System,” Journal of Neuroscience 37, no. 9 (2017): 2395–2402, 10.1523/JNEUROSCI.2754-16.2017. 28137972 PMC6596847

[11] S. Dreha‐Kulaczewski , A. A. Joseph , K. D. Merboldt , H. C. Ludwig , J. Gärtner , and J. Frahm , “Inspiration Is the Major Regulator of Human CSF Flow,” Journal of Neuroscience 35, no. 6 (2015): 2485–2491, 10.1523/JNEUROSCI.3246-14.2015. 25673843 PMC6605608

[12] G. Ringstad and P. K. Eide , “Cerebrospinal Fluid Tracer Efflux to Parasagittal Dura in Humans,” Nature Communications 11, no. 1 (2020): 1–9. 10.1038/s41467-019-14195-x PMC6969040 31953399

[13] M. E. Wagshul , P. K. Eide , and J. R. Madsen , “The Pulsating Brain: A Review of Experimental and Clinical Studies of Intracranial Pulsatility,” Fluids and Barriers of the CNS 8 (2011): 5, 10.1186/2045-8118-8-5. 21349153 PMC3042979

[14] S. Yildiz , J. Grinstead , A. Hildebrand , et al., “Immediate Impact of Yogic Breathing on Pulsatile Cerebrospinal Fluid Dynamics,” Scientific Reports 12, no. 1 (2022): 10894, 10.1038/s41598-022-15034-8. 35764793 PMC9240010

[15] N. Huotari , L. Raitamaa , H. Helakari , et al., “Sampling Rate Effects on Resting State fMRI Metrics,” Frontiers in Neuroscience 13 (2019): 279, 10.3389/fnins.2019.00279. 31001071 PMC6454039

[16] C. Windischberger , H. Langenberger , T. Sycha , et al., “On the Origin of Respiratory Artifacts in BOLD‐EPI of the Human Brain,” Magnetic Resonance Imaging 20, no. 8 (2002): 575–582, 10.1016/S0730-725X(02)00563-5. 12467863

[17] P. T. Fox and M. E. Raichle , “Focal Physiological Uncoupling of Cerebral Blood Flow and Oxidative Metabolism During Somatosensory Stimulation in Human Subjects,” Proceedings of the National Academy of Sciences of the United States of America 83, no. 4 (1986): 1140–1144. 3485282 10.1073/pnas.83.4.1140 PMC323027

[18] L. Pauling and C. D. Coryell , “The Magnetic Properties and Structure of Hemoglobin, Oxyhemoglobin and Carbonmonoxyhemoglobin,” Proceedings of the National Academy of Sciences of the United States of America 22, no. 4 (1936): 210–216, 10.1073/pnas.22.4.210. 16577697 PMC1076743

[19] S. Ogawa , T. M. Lee , A. R. Kay , and D. W. Tank , “Brain Magnetic Resonance Imaging With Contrast Dependent on Blood Oxygenation,” Proceedings of the National Academy of Sciences of the United States of America 87, no. 24 (1990): 9868–9872. 2124706 10.1073/pnas.87.24.9868 PMC55275

[20] P. A. Bandettini , E. C. Wong , R. S. Hinks , R. S. Tikofsky , and J. S. Hyde , “Time Course EPI of Human Brain Function During Task Activation,” Magnetic Resonance in Medicine 25, no. 2 (1992): 390–397, 10.1002/mrm.1910250220. 1614324

[21] K. K. Kwong , J. W. Belliveau , D. A. Chesler , et al., “Dynamic Magnetic Resonance Imaging of Human Brain Activity During Primary Sensory Stimulation,” Proceedings of the National Academy of Sciences of the United States of America 89, no. 12 (1992): 5675–5679. 1608978 10.1073/pnas.89.12.5675 PMC49355

[22] S. Ogawa , D. W. Tank , R. Menon , et al., “Intrinsic Signal Changes Accompanying Sensory Stimulation: Functional Brain Mapping With Magnetic Resonance Imaging,” Proceedings of the National Academy of Sciences of the United States of America 89, no. 13 (1992): 5951–5955. 1631079 10.1073/pnas.89.13.5951 PMC402116

[23] P. J. Drew , A. Y. Shih , and D. Kleinfeld , “Fluctuating and Sensory‐Induced Vasodynamics in Rodent Cortex Extend Arteriole Capacity,” Proceedings of the National Academy of Sciences of the United States of America 108, no. 20 (2011): 8473–8478, 10.1073/pnas.1100428108. 21536897 PMC3100929

[24] S. Grubb , C. Cai , B. O. Hald , et al., “Precapillary Sphincters Maintain Perfusion in the Cerebral Cortex,” Nature Communications 11 (2020): 395, 10.1038/s41467-020-14330-z. PMC6971292 31959752

[25] N. Huotari , J. Tuunanen , L. Raitamaa , et al., “Cardiovascular Pulsatility Increases in Visual Cortex Before Blood Oxygen Level Dependent Response During Stimulus,” Frontiers in Neuroscience 16 (2022): 836378, 10.3389/fnins.2022.836378. 35185462 PMC8853630

[26] Y. Ma , M. A. Shaik , M. G. Kozberg , et al., “Resting‐State Hemodynamics Are Spatiotemporally Coupled to Synchronized and Symmetric Neural Activity in Excitatory Neurons,” Proceedings of the National Academy of Sciences of the United States of America 113, no. 52 (2016): E8463–E8471, 10.1073/pnas.1525369113. 27974609 PMC5206542

[27] B. B. Biswal , A. P. Pathak , J. L. Ulmer , and A. G. Hudetz , “Decoupling of the Hemodynamic and Activation‐Induced Delays in Functional Magnetic Resonance Imaging,” Journal of Computer Assisted Tomography 27, no. 2 (2003): 219–225. 12703015 10.1097/00004728-200303000-00019

[28] R. B. Buxton , “Dynamic Models of BOLD Contrast,” NeuroImage 62, no. 2 (2012): 953–961, 10.1016/j.neuroimage.2012.01.012. 22245339 PMC3545646

[29] J. Hennig , V. Kiviniemi , B. Riemenschneider , et al., “15 Years MR‐Encephalography,” Magnetic Resonance Materials in Physics, Biology and Medicine 34, no. 1 (2021): 85–108, 10.1007/s10334-020-00891-z. PMC7910380 33079327

[30] F. H. Lin , K. W. K. Tsai , Y. H. Chu , et al., “Ultrafast Inverse Imaging Techniques for fMRI,” NeuroImage 62, no. 2 (2012): 699–705. 22285221 10.1016/j.neuroimage.2012.01.072 PMC3377851

[31] S. Posse , E. Ackley , R. Mutihac , et al., “High‐Speed Real‐Time Resting‐State FMRI Using Multi‐Slab Echo‐Volumar Imaging,” Frontiers in Human Neuroscience 7 (2013): 479, 10.3389/fnhum.2013.00479. 23986677 PMC3752525

[32] S. J. van Veluw , S. S. Hou , M. Calvo‐Rodriguez , et al., “Vasomotion as a Driving Force for Paravascular Clearance in the Awake Mouse Brain,” Neuron 105, no. 3 (2020): 549–561.e5. 31810839 10.1016/j.neuron.2019.10.033 PMC7028316

[33] A. K. Diem , R. O. Carare , R. O. Weller , and N. W. Bressloff , “A Control Mechanism for Intra‐Mural Peri‐Arterial Drainage via Astrocytes: How Neuronal Activity Could Improve Waste Clearance From the Brain,” PLoS ONE 13, no. 10 (2018): e0205276. 30286191 10.1371/journal.pone.0205276 PMC6171921

[34] L. Bojarskaite , A. Vallet , D. M. Bjørnstad , et al., “Sleep Cycle‐Dependent Vascular Dynamics in Male Mice and the Predicted Effects on Perivascular Cerebrospinal Fluid Flow and Solute Transport,” Nature Communications 14 (2023): 953, 10.1038/s41467-023-36643-5. PMC9941497 36806170

[35] V. Kiviniemi , “6–Physiological Brain Pulsations,” in Advances in Resting‐State Functional MRI. Neuroimaging Methods and Applications, eds. J. Chen and C. Chang (Academic Press, 2023), 131–153, 10.1016/B978-0-323-91688-2.00012-6.

[36] J. C. Kucewicz , B. Dunmire , D. F. Leotta , H. Panagiotides , M. Paun , and K. W. Beach , “Functional Tissue Pulsatility Imaging of the Brain During Visual Stimulation,” Ultrasound in Medicine & Biology 33, no. 5 (2007): 681–690, 10.1016/j.ultrasmedbio.2006.11.008. 17346872 PMC1995427

[37] J. H. Duyn , “Steady State Effects in Fast Gradient Echo Magnetic Resonance Imaging,” Magnetic Resonance in Medicine 37, no. 4 (1997): 559–568, 10.1002/mrm.1910370414. 9094078

[38] G. K. von Schulthess and C. B. Higgins , “Blood Flow Imaging With MR: Spin‐Phase Phenomena,” Radiology 157, no. 3 (1985): 687–695, 10.1148/radiology.157.3.2997836. 2997836

[39] J. J. Iliff , M. Wang , D. M. Zeppenfeld , et al., “Cerebral Arterial Pulsation Drives Paravascular CSF‐Interstitial Fluid Exchange in the Murine Brain,” Journal of Neuroscience 33, no. 46 (2013): 18190–18199, 10.1523/JNEUROSCI.1592-13.2013. 24227727 PMC3866416

[40] M. Nedergaard , “Neuroscience. Garbage Truck of the Brain,” Science 340, no. 6140 (2013): 1529–1530, 10.1126/science.1240514. 23812703 PMC3749839

[41] T. Nakano , R. Tominaga , I. Nagano , H. Okabe , and H. Yasui , “Pulsatile Flow Enhances Endothelium‐Derived Nitric Oxide Release in the Peripheral Vasculature,” American Journal of Physiology‐Heart and Circulatory Physiology 278, no. 4 (2000): H1098–H1104, 10.1152/ajpheart.2000.278.4.H1098. 10749703

[42] L. Xie , H. Kang , Q. Xu , et al., “Sleep Drives Metabolite Clearance From the Adult Brain,” Science 342, no. 6156 (2013): 373–377, 10.1126/science.1241224. 24136970 PMC3880190

[43] R. M. Birn , K. Murphy , D. A. Handwerker , and P. A. Bandettini , “fMRI in the Presence of Task‐Correlated Breathing Variations,” NeuroImage 47, no. 3 (2009): 1092–1104, 10.1016/j.neuroimage.2009.05.030. 19460443 PMC2998293

[44] R. G. Wise , K. Ide , M. J. Poulin , and I. Tracey , “Resting Fluctuations in Arterial Carbon Dioxide Induce Significant Low Frequency Variations in BOLD Signal,” NeuroImage 21, no. 4 (2004): 1652–1664, 10.1016/j.neuroimage.2003.11.025. 15050588

[45] H. Yuan , V. Zotev , R. Phillips , and J. Bodurka , “Correlated Slow Fluctuations in Respiration, EEG, and BOLD fMRI,” NeuroImage 79 (2013): 81–93, 10.1016/j.neuroimage.2013.04.068. 23631982

[46] N. E. Fultz , G. Bonmassar , K. Setsompop , et al., “Coupled Electrophysiological, Hemodynamic, and Cerebrospinal Fluid Oscillations in Human Sleep,” Science 366, no. 6465 (2019): 628–631. 31672896 10.1126/science.aax5440 PMC7309589

[47] S. Yamada , M. Miyazaki , Y. Yamashita , et al., “Influence of Respiration on Cerebrospinal Fluid Movement Using Magnetic Resonance Spin Labeling,” Fluids and Barriers of the CNS 10, no. 1 (2013): 36. 24373186 10.1186/2045-8118-10-36 PMC3895787

[48] M. Matsumae , K. Kuroda , S. Yatsushiro , et al., “Changing the Currently Held Concept of Cerebrospinal Fluid Dynamics Based on Shared Findings of Cerebrospinal Fluid Motion in the Cranial Cavity Using Various Types of Magnetic Resonance Imaging Techniques,” Neurologia Medico‐Chirurgica 59, no. 4 (2019): 133–146, 10.2176/nmc.ra.2018-0272. 30814424 PMC6465527

[49] Y. Wang , P. van Gelderen , J. A. de Zwart , et al., “Cerebrovascular Activity Is a Major Factor in the Cerebrospinal Fluid Flow Dynamics,” NeuroImage 258 (2022): 119362, 10.1016/j.neuroimage.2022.119362. 35688316 PMC9271599

[50] V. Vinje , G. Ringstad , E. K. Lindstrøm , et al., “Respiratory Influence on Cerebrospinal Fluid Flow—A Computational Study Based on Long‐Term Intracranial Pressure Measurements,” Scientific Reports 9, no. 1 (2019): 1–13. 31278278 10.1038/s41598-019-46055-5 PMC6611841

[51] J. Jukkola , M. Kaakinen , A. Singh , et al., “Blood Pressure Lowering Enhances Cerebrospinal Fluid Efflux to the Systemic Circulation Primarily via the Lymphatic Vasculature,” Fluids and Barriers of the CNS 21, no. 1 (2024): 12, 10.1186/s12987-024-00509-9. 38279178 PMC10821255

[52] P. K. Eide and G. Ringstad , “MRI With Intrathecal MRI Gadolinium Contrast Medium Administration: A Possible Method to Assess Glymphatic Function in Human Brain,” Acta Radiol Open 4, no. 11 (2015): 2058460115609635, 10.1177/2058460115609635. 26634147 PMC4652208

[53] P. J. Drew , C. Mateo , K. L. Turner , X. Yu , and D. Kleinfeld , “Ultra‐Slow Oscillations in fMRI and Resting‐State Connectivity: Neuronal and Vascular Contributions and Technical Confounds,” Neuron 107, no. 5 (2020): 782–804, 10.1016/j.neuron.2020.07.020. 32791040 PMC7886622

[54] V. Kiviniemi , Spontaneous Blood Oxygen Fluctuation in Awake and Sedated Brain Cortex—A Bold fMRI Study. jultika.oulu.fi. June 18, 2004, accessed February 6, 2024, https://oulurepo.oulu.fi/handle/10024/34634.

[55] K. Miakawa , H. P. Koepchen , and C. Polosa , Mechanism of Blood Pressure Waves (Springer‐Verlag, 1984).

[56] M. Intaglietta , Vasomotion and Flowmotion: Physiological Mechanisms and Clinical Evidence (1990), accessed February 6, 2024, https://journals.sagepub.com/doi/10.1177/1358836X9000100202.

[57] B. Biswal , F. Z. Yetkin , V. M. Haughton , and J. S. Hyde , “Functional Connectivity in the Motor Cortex of Resting Human Brain Using Echo‐Planar MRI,” Magnetic Resonance in Medicine 34, no. 4 (1995): 537–541. 8524021 10.1002/mrm.1910340409

[58] V. Kiviniemi , J. Jauhiainen , O. Tervonen , et al., “Slow Vasomotor Fluctuation in fMRI of Anesthetized Child Brain,” Magnetic Resonance in Medicine 44, no. 3 (2000): 373–378. 10975887 10.1002/1522-2594(200009)44:3<373::aid-mrm5>3.0.co;2-p

[59] C. F. Beckmann and S. M. Smith , “Probabilistic Independent Component Analysis for Functional Magnetic Resonance Imaging,” IEEE Transactions on Medical Imaging 23, no. 2 (2004): 137–152. 14964560 10.1109/TMI.2003.822821

[60] M. D. Fox and M. E. Raichle , “Spontaneous Fluctuations in Brain Activity Observed With Functional Magnetic Resonance Imaging,” Nature Reviews Neuroscience 8, no. 9 (2007): 700–711, 10.1038/nrn2201. 17704812

[61] V. Kiviniemi , J. H. Kantola , J. Jauhiainen , A. Hyvärinen , and O. Tervonen , “Independent Component Analysis of Nondeterministic fMRI Signal Sources,” NeuroImage 19, no. 2 (2003): 253–260. 12814576 10.1016/s1053-8119(03)00097-1

[62] S. M. Smith , P. T. Fox , K. L. Miller , et al., “Correspondence of the Brain's Functional Architecture During Activation and Rest,” Proceedings of the National Academy of Sciences of the United States of America 106, no. 31 (2009): 13040–13045. 19620724 10.1073/pnas.0905267106 PMC2722273

[63] T. Bolt , J. S. Nomi , D. Bzdok , et al., “A Parsimonious Description of Global Functional Brain Organization in Three Spatiotemporal Patterns,” Nature Neuroscience 25, no. 8 (2022): 1093–1103, 10.1038/s41593-022-01118-1. 35902649 PMC13716133

[64] V. Kiviniemi , T. Starck , J. Remes , et al., “Functional Segmentation of the Brain Cortex Using High Model Order Group PICA,” Human Brain Mapping 30, no. 12 (2009): 3865–3886, 10.1002/hbm.20813. 19507160 PMC6870574

[65] A. Abbas , M. Belloy , A. Kashyap , et al., “Quasi‐Periodic Patterns Contribute to Functional Connectivity in the Brain,” NeuroImage 191 (2019): 193–204, 10.1016/j.neuroimage.2019.01.076. 30753928 PMC6440826

[66] Z. Rajna , L. Raitamaa , T. Tuovinen , J. Heikkila , V. Kiviniemi , and T. Seppanen , “3D Multi‐Resolution Optical Flow Analysis of Cardiovascular Pulse Propagation in Human Brain,” IEEE Transactions on Medical Imaging 38, no. 9 (2019): 2028–2036, 10.1109/TMI.2019.2904762. 30892202

[67] Z. Rajna , J. Kananen , A. Keskinarkaus , T. Seppänen , and V. Kiviniemi , “Detection of Short‐Term Activity Avalanches in Human Brain Default Mode Network With Ultrafast MR Encephalography,” Frontiers in Human Neuroscience 9 (2015): 448, 10.3389/fnhum.2015.00448. 26321936 PMC4531800

[68] J. C. Gore , M. Li , Y. Gao , et al., “Functional MRI and Resting State Connectivity in White Matter—A Mini‐Review,” Magnetic Resonance Imaging 63 (2019): 1–11, 10.1016/j.mri.2019.07.017. 31376477 PMC6861686

[69] N. K. Logothetis , J. Pauls , M. Augath , T. Trinath , and A. Oeltermann , “Neurophysiological Investigation of the Basis of the fMRI Signal,” Nature 412, no. 6843 (2001): 150–157. 11449264 10.1038/35084005

[70] Y. Ma , M. A. Shaik , S. H. Kim , et al., “Wide‐Field Optical Mapping of Neural Activity and Brain Haemodynamics: Considerations and Novel Approaches,” Philosophical Transactions of the Royal Society B 371 (2016): 20150360, 10.1098/rstb.2015.0360. PMC5003860 27574312

[71] V. Kiviniemi , H. Haanpää , J. H. Kantola , et al., “Midazolam Sedation Increases Fluctuation and Synchrony of the Resting Brain BOLD Signal,” Magnetic Resonance Imaging 23, no. 4 (2005): 531–537, 10.1016/j.mri.2005.02.009. 15919598

[72] C. Chang , D. A. Leopold , M. L. Scholvinck , et al., “Tracking Brain Arousal Fluctuations With fMRI,” Proceedings of the National Academy of Sciences of the United States of America 113, no. 16 (2016): 4518–4523, 10.1073/pnas.1520613113. 27051064 PMC4843437

[73] X. Liu , J. A. de Zwart , M. L. Schölvinck , et al., “Subcortical Evidence for a Contribution of Arousal to fMRI Studies of Brain Activity,” Nature Communications 9, no. 1 (2018): 395. 10.1038/s41467-017-02815-3 PMC5786066 29374172

[74] H. M. Chow , S. G. Horovitz , W. S. Carr , et al., “Rhythmic Alternating Patterns of Brain Activity Distinguish Rapid Eye Movement Sleep From Other States of Consciousness,” Proceedings of the National Academy of Sciences of the United States of America 110, no. 25 (2013): 10300–10305, 10.1073/pnas.1217691110. 23733938 PMC3690889

[75] M. Fukunaga , S. G. Horovitz , P. van Gelderen , et al., “Large‐Amplitude, Spatially Correlated Fluctuations in BOLD fMRI Signals During Extended Rest and Early Sleep Stages,” Magnetic Resonance Imaging 24, no. 8 (2006): 979–992. 16997067 10.1016/j.mri.2006.04.018

[76] S. G. Horovitz , M. Fukunaga , J. A. de Zwart , et al., “Low Frequency BOLD Fluctuations During Resting Wakefulness and Light Sleep: A Simultaneous EEG‐fMRI Study,” Human Brain Mapping 29, no. 6 (2008): 671–682, 10.1002/hbm.20428. 17598166 PMC6871022

[77] P. S. Özbay , C. Chang , D. Picchioni , et al., “Sympathetic Activity Contributes to the fMRI Signal,” Communications Biology 2 (2019): 421, 10.1038/s42003-019-0659-0. 31754651 PMC6861267

[78] D. Picchioni , P. S. Özbay , H. Mandelkow , et al., “Autonomic Arousals Contribute to Brain Fluid Pulsations During Sleep,” NeuroImage 249 (2022): 118888, 10.1016/j.neuroimage.2022.118888. 35017126 PMC11395500

[79] F. Ding , J. O'Donnell , Q. Xu , N. Kang , N. Goldman , and M. Nedergaard , “Changes in the Composition of Brain Interstitial Ions Control the Sleep‐Wake Cycle,” Science 352, no. 6285 (2016): 550–555, 10.1126/science.aad4821. 27126038 PMC5441687

[80] H. Helakari , M. Järvelä , T. Väyrynen , et al., “Effect of Sleep Deprivation and NREM Sleep Stage on Physiological Brain Pulsations,” Frontiers in Neuroscience 17 (2023): 1275184, 10.3389/fnins.2023.1275184. 38105924 PMC10722275

[81] H. Helakari , V. Korhonen , S. C. Holst , et al., “Human NREM Sleep Promotes Brain‐Wide Vasomotor and Respiratory Pulsations,” Journal of Neuroscience 42, no. 12 (2022): 2503–2515, 10.1523/JNEUROSCI.0934-21.2022. 35135852 PMC8944230

[82] P. Mahon , B. R. Greene , E. M. Lynch , B. McNamara , and G. D. Shorten , “Can State or Response Entropy Be Used as a Measure of Sleep Depth?,” Anaesthesia 63, no. 12 (2008): 1309–1313. 19032298 10.1111/j.1365-2044.2008.05675.x

[83] V. Malik , D. Smith , and T. Lee‐Chiong , “Respiratory Physiology During Sleep,” Sleep Medicine Clinics 7, no. 3 (2012): 497–505, 10.1016/j.jsmc.2012.06.011.

[84] M. Sowho , J. Amatoury , J. P. Kirkness , and S. P. Patil , “Sleep and Respiratory Physiology in Adults,” Clinics in Chest Medicine 35, no. 3 (2014): 469–481, 10.1016/j.ccm.2014.06.002. 25156763

[85] J. Gisolf , J. J. Van Lieshout , K. Van Heusden , F. Pott , W. J. Stok , and J. M. Karemaker , “Human Cerebral Venous Outflow Pathway Depends on Posture and Central Venous Pressure,” Journal of Physiology 560, no. Pt 1 (2004): 317–327, 10.1113/jphysiol.2004.070409. 15284348 PMC1665206

[86] J. Tuunanen , H. Helakari , N. Huotari , et al., “Cardiovascular and Vasomotor Pulsations in the Brain and Periphery During Awake and NREM Sleep in a Multimodal fMRI Study,” Frontiers in Neuroscience 18 (2024): 1457732, 10.3389/fnins.2024.1457732. 39440186 PMC11493778

[87] A. Elabasy , H. Helakari , T. Väyrynen , et al., Sleep Increases Propagation Speed of Physiological Brain Pulsations, published January 27, 2025, 10.1101/2025.01.09.632145.

[88] T. Väyrynen , J. Tuunanen , H. Helakari , et al., Sleep‐Induced Vasomotor Pulsation Is a Driver of Cerebrospinal Fluid and Blood‐Brain Barrier Dynamics in the Human Brain, published October 18, 2024, 10.1101/2024.10.16.618430.

[89] N. L. Hauglund , M. Andersen , K. Tokarska , et al., “Norepinephrine‐Mediated Slow Vasomotion Drives Glymphatic Clearance During Sleep,” Cell 188, no. 3 (2025): 606–622.e17, 10.1016/j.cell.2024.11.027. 39788123 PMC12340670

[90] J. Kananen , H. Helakari , V. Korhonen , et al., “Respiratory‐Related Brain Pulsations Are Increased in Epilepsy—A Two‐Centre Functional MRI Study,” Brain Communications 2, no. 2 (2020): fcaa076. 32954328 10.1093/braincomms/fcaa076 PMC7472909

[91] M. Järvelä , J. Kananen , V. Korhonen , N. Huotari , H. Ansakorpi , and V. Kiviniemi , “Increased Very Low Frequency Pulsations and Decreased Cardiorespiratory Pulsations Suggest Altered Brain Clearance in Narcolepsy,” Communications Medicine 2, no. 1 (2022): 1–13, 10.1038/s43856-022-00187-4. 36193214 PMC9525269

[92] M. Järvelä , J. Kananen , H. Helakari , et al., Arousal State Control of Physiological Human Brain Pulsations, published January 28, 2025, 10.1101/2025.01.27.635032.

[93] V. Poltojainen , J. Kemppainen , N. Keinänen , et al., “Physiological Instability Is Linked to Mortality in Primary Central Nervous System Lymphoma: A Case–Control fMRI Study,” Human Brain Mapping 43, no. 13 (2022): 4030–4044, 10.1002/hbm.25901. 35543292 PMC9374894

[94] P. A. Bandettini , A. Jesmanowicz , E. C. Wong , and J. S. Hyde , “Processing Strategies for Time‐Course Data Sets in Functional MRI of the Human Brain,” Magnetic Resonance in Medicine 30, no. 2 (1993): 161–173, 10.1002/mrm.1910300204. 8366797

[95] Z. Yu‐Feng , H. Yong , Z. Chao‐Zhe , et al., “Altered Baseline Brain Activity in Children With ADHD Revealed by Resting‐State Functional MRI,” Brain & Development 29, no. 2 (2007): 83–91, 10.1016/j.braindev.2006.07.002. 16919409

[96] B. B. Biswal , M. Mennes , X. N. Zuo , et al., “Toward Discovery Science of Human Brain Function,” Proceedings of the National Academy of Sciences of the United States of America 107, no. 10 (2010): 4734–4739, 10.1073/pnas.0911855107. 20176931 PMC2842060

[97] D. Mao , Z. Ding , W. Jia , et al., “Low‐Frequency Fluctuations of the Resting Brain: High Magnitude Does Not Equal High Reliability,” PLoS ONE 10, no. 6 (2015): e0128117, 10.1371/journal.pone.0128117. 26053265 PMC4460034

[98] W. Peng , T. M. Achariyar , B. Li , et al., “Suppression of Glymphatic Fluid Transport in a Mouse Model of Alzheimer's Disease,” Neurobiology of Disease 93 (2016): 215–225, 10.1016/j.nbd.2016.05.015. 27234656 PMC4980916

[99] H. Jahanian , W. W. Ni , T. Christen , M. E. Moseley , M. K. Tamura , and G. Zaharchuk , “Spontaneous Bold Signal Fluctuations in Young Healthy Subjects and Elderly Patients With Chronic Kidney Disease,” PLoS ONE 9, no. 3 (2014): e92539, 10.1371/journal.pone.0092539. 24651703 PMC3961376

[100] I. Makedonov , S. E. Black , and B. J. Macintosh , “BOLD fMRI in the White Matter as a Marker of Aging and Small Vessel Disease,” PLoS ONE 8, no. 7 (2013): e67652, 10.1371/journal.pone.0067652. 23844047 PMC3699638

[101] I. Makedonov , S. E. Black , and B. J. MacIntosh , “Cerebral Small Vessel Disease in Aging and Alzheimer's Disease: A Comparative Study Using MRI and SPECT,” European Journal of Neurology 20, no. 2 (2013): 243–250. 22742818 10.1111/j.1468-1331.2012.03785.x

[102] I. Makedonov , J. J. Chen , M. Masellis , B. J. MacIntosh , and Alzheimer's Disease Neuroimaging Initiative , “Physiological Fluctuations in White Matter Are Increased in Alzheimer's Disease and Correlate With Neuroimaging and Cognitive Biomarkers,” Neurobiology of Aging 37 (2016): 12–18, 10.1016/j.neurobiolaging.2015.09.010. 26476600

[103] T. Tuovinen , J. Häkli , R. Rytty , et al., “The Relative Brain Signal Variability Increases in the Behavioral Variant of Frontotemporal Dementia and Alzheimer's Disease but not in Schizophrenia,” Journal of Cerebral Blood Flow and Metabolism 44, no. 12 (2024): 1535–1549, 10.1177/0271678X241262583. 38897598 PMC11574935

[104] T. Tuovinen , J. Kananen , Z. Rajna , et al., “The Variability of Functional MRI Brain Signal Increases in Alzheimer's Disease at Cardiorespiratory Frequencies,” Scientific Reports 10, no. 1 (2020): 1–11. 33298996 10.1038/s41598-020-77984-1 PMC7726142

[105] Z. Rajna , H. Mattila , N. Huotari , et al., “Cardiovascular Brain Impulses in Alzheimer's Disease,” Brain 144 (2021): 2214–2226. 33787890 10.1093/brain/awab144 PMC8422353

[106] D. A. Nation , M. D. Sweeney , A. Montagne , et al., “Blood–Brain Barrier Breakdown Is an Early Biomarker of Human Cognitive Dysfunction,” Nature Medicine 25, no. 2 (2019): 270–276. 10.1038/s41591-018-0297-y PMC6367058 30643288

[107] C. D. Morrone , J. Bishay , and J. McLaurin , “Potential Role of Venular Amyloid in Alzheimer's Disease Pathogenesis,” International Journal of Molecular Sciences 21, no. 6 (2020): 1985, 10.3390/ijms21061985. 32183293 PMC7139584

[108] M. Arbel‐Ornath , E. Hudry , K. Eikermann‐Haerter , et al., “Interstitial Fluid Drainage Is Impaired in Ischemic Stroke and Alzheimer's Disease Mouse Models,” Acta Neuropathologica 126, no. 3 (2013): 353–364. 23818064 10.1007/s00401-013-1145-2 PMC3810119

[109] R. Hussain , J. Tithof , W. Wang , et al., “Potentiating Glymphatic Drainage Minimizes Post‐Traumatic Cerebral Oedema,” Nature 623, no. 7989 (2023): 992–1000, 10.1038/s41586-023-06737-7. 37968397 PMC11216305

[110] A. Hussein , J. L. Matthews , C. Syme , et al., “The Association Between Resting‐State Functional Magnetic Resonance Imaging and Aortic Pulse‐Wave Velocity in Healthy Adults,” Human Brain Mapping 41, no. 8 (2020): 2121–2135, 10.1002/hbm.24934. 32034832 PMC7268071

[111] M. Nedergaard and S. A. Goldman , “Glymphatic Failure as a Final Common Pathway to Dementia,” Science 370, no. 6512 (2020): 50–56. 33004510 10.1126/science.abb8739 PMC8186542

[112] D. M. Zeppenfeld , M. Simon , J. D. Haswell , et al., “Association of Perivascular Localization of Aquaporin‐4 With Cognition and Alzheimer Disease in Aging Brains,” JAMA Neurology 74, no. 1 (2017): 91–99, 10.1001/jamaneurol.2016.4370. 27893874

[113] T. E. Scammell , “Narcolepsy,” New England Journal of Medicine 373 (2015): 2654–2662. 26716917 10.1056/NEJMra1500587

[114] M. Järvelä , V. Raatikainen , A. Kotila , et al., “Lag Analysis of Fast fMRI Reveals Delayed Information Flow Between the Default Mode and Other Networks in Narcolepsy,” Cerebral Cortex Communications 1, no. 1 (2020): tgaa073. 34296133 10.1093/texcom/tgaa073 PMC8153076

[115] T. Eid , T. S. Lee , M. J. Thomas , et al., “Loss of Perivascular Aquaporin 4 May Underlie Deficient Water and K+ Homeostasis in the Human Epileptogenic Hippocampus,” Proceedings of the National Academy of Sciences of the United States of America 102, no. 4 (2005): 1193–1198. 15657133 10.1073/pnas.0409308102 PMC545857

[116] O. Devinsky , A. Vezzani , S. Najjar , N. C. De Lanerolle , and M. A. Rogawski , “Glia and Epilepsy: Excitability and Inflammation,” Trends in Neurosciences 36, no. 3 (2013): 174–184, 10.1016/j.tins.2012.11.008. 23298414

[117] J. L. Herrero , S. Khuvis , E. Yeagle , M. Cerf , and A. D. Mehta , “Breathing Above the Brain Stem: Volitional Control and Attentional Modulation in Humans,” Journal of Neurophysiology 119, no. 1 (2018): 145–159. 28954895 10.1152/jn.00551.2017 PMC5866472

[118] C. Zelano , H. Jiang , G. Zhou , et al., “Nasal Respiration Entrains Human Limbic Oscillations and Modulates Cognitive Function,” Journal of Neuroscience 36, no. 49 (2016): 12448–12467, 10.1523/JNEUROSCI.2586-16.2016. 27927961 PMC5148230

[119] J. Kananen , Increased Effect of Physiological Respiratory Brain Pulsations in Focal‐Onset Epilepsy.

[120] J. Kananen , T. Tuovinen , H. Ansakorpi , et al., “Altered Physiological Brain Variation in Drug‐Resistant Epilepsy,” Brain and Behavior: A Cognitive Neuroscience Perspective 8, no. 9 (2018): e01090, 10.1002/brb3.1090. PMC6160661 30112813

[121] J. Kananen , M. Järvelä , V. Korhonen , et al., “Increased Interictal Synchronicity of Respiratory Related Brain Pulsations in Epilepsy,” Journal of Cerebral Blood Flow and Metabolism 42 (2022): 0271678X221099703, 10.1177/0271678X221099703. PMC9536129 35570730

[122] A. Elabasy , M. Suhonen , Z. Rajna , et al., “Respiratory Brain Impulse Propagation in Focal Epilepsy,” Scientific Reports 13 (2023): 5222, 10.1038/s41598-023-32271-7. 36997658 PMC10063583

[123] J. Gonzalez‐Castillo , V. Roopchansingh , P. A. Bandettini , and J. Bodurka , “Physiological Noise Effects on the Flip Angle Selection in BOLD fMRI,” NeuroImage 54, no. 4 (2011): 2764–2778, 10.1016/j.neuroimage.2010.11.020. 21073963 PMC3020268

[124] J. Assländer , B. Zahneisen , T. Hugger , et al., “Single Shot Whole Brain Imaging Using Spherical Stack of Spirals Trajectories,” NeuroImage 73 (2013): 59–70, 10.1016/j.neuroimage.2013.01.065. 23384526

[125] S. M. Ebrahimi , J. Tuunanen , V. Saarela , et al., “Synchronous Functional Magnetic Resonance Eye Imaging, Video Ophthalmoscopy, and Eye Surface Imaging Reveal the Human Brain and Eye Pulsation Mechanisms,” Scientific Reports 14, no. 1 (2024): 2250, 10.1038/s41598-023-51069-1. 38278832 PMC10817967

[126] V. Raatikainen , “Dynamic Lag Analysis of Human Brain Activity Propagation: A Fast fMRI Study,” (thesis, Acta Universtas Ouluensis, 2021), https://urn.fi/URN:ISBN:9789526229234.

[127] V. Raatikainen , V. Korhonen , V. Borchardt , et al., “Dynamic Lag Analysis Reveals Atypical Brain Information Flow in Autism Spectrum Disorder,” Autism Research 13, no. 2 (2020): 244–258, 10.1002/aur.2218. 31637863 PMC7027814

[128] V. Raatikainen , N. Huotari , V. Korhonen , et al., “Combined Spatiotemporal ICA (stICA) for Continuous and Dynamic Lag Structure Analysis of MREG Data,” NeuroImage 148 (2017): 352–363. 28088482 10.1016/j.neuroimage.2017.01.024

